# Estimation of breed composition of South African sheep affected with wet carcass syndrome 

**DOI:** 10.3389/fgene.2025.1635947

**Published:** 2025-07-21

**Authors:** Bhaveni B. Kooverjee, Magrieta A. Van Der Nest, Michael D. MacNeil, Michiel M. Scholtz, Frederick W. C. Neser, Pranisha Soma

**Affiliations:** ^1^ Department of Animal Breeding and Genetics, Agricultural Research Council – Animal Production, Irene, South Africa; ^2^ Department of Animal Sciences, University of the Free State, Bloemfontein, South Africa; ^3^ Forestry and Agricultural Biotechnology Institute (FABI), University of Pretoria, Pretoria, South Africa; ^4^ Delta G, Miles City, MT, United States

**Keywords:** case-control study, meat quality, breed specificity, population genomics, ovine genetics, genetic admixture

## Abstract

Wet carcass syndrome (WCS), a condition that negatively affects the quality of carcasses after slaughter, is seriously threatening the South African sheep industry. Despite its economic impact, the underlying genetic mechanisms of WCS remain unknown. Initially, WCS was predominantly observed in Dorper sheep, leading to speculation that the condition was breed-specific. However, recent reports indicate WCS has occurred in various sheep breeds. This study aimed to determine whether WCS is breed-specific and whether breed composition influences its incidence. Meat samples from 164 WCS-affected and 83 unaffected sheep were collected and genotyped using the Ovine 50K SNP Bead Chip. Principal Component Analysis (PCA) and ancestry matrix assessments revealed that WCS-affected and unaffected sheep belonged to different commercial breeds. Additionally, crossbred animals were affected. These findings suggest that WCS is strongly associated with breed and that Dorper and Merino-types show heightened susceptibility. The genetic diversity of the affected animals suggests a multifactorial etiology, potentially involving environmental and managerial factors. Future studies should also explore the physiological mechanisms underlying WCS, including metabolic and stress-related pathways, to develop effective prevention strategies.

## 1 Introduction

**FIGURE 1:**
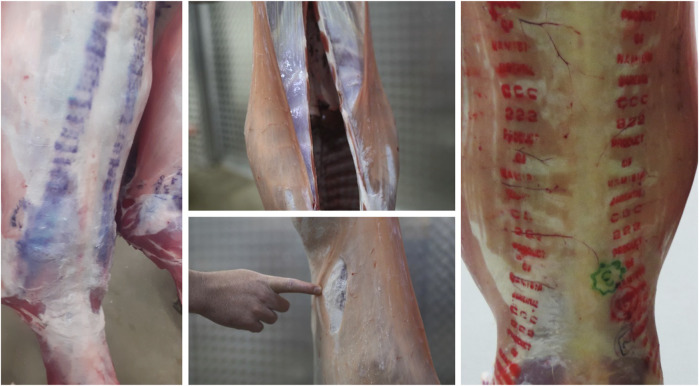
Images of the carcasses showing the presence of wet carcass syndrome. Grading marks unrecognizable due to distortion by fluid (Left), Thick transparent fluid coating the carcass (Middle-top), Uppermost layer of skin can be pulled away due to fluid build-up under the skin (Middle-bottom), An unaffected carcass with clear grading marks (Right).

The sheep industry is the third-largest contributor to the South African agricultural economy. South African sheep farming is majorly predominant in Eastern Cape (30%), Northern Cape (24%), and Free State (20%) while to lesser extent in Western Cape (∼12%) and Mpumalanga (∼7%) and minimal in the other four provinces (∼7%) (Department of Agriculture, Land, Reform and Rural Development, 2024). In 2022, sheep meat production in South Africa reached approximately 4.81 million head, surpassing pig and cattle production, which were estimated at 3.68 million and 3.26 million head, respectively (Cowling, 2024). Over the past decade, the industry has shown increasing economic benefits through the production of meat, wool, milk, and other by-products (Molotsi et al., 2017). However, wet carcass syndrome (WCS), a condition that negatively impacts carcass quality, poses a significant challenge to the sheep industry. The National Animal Health Disease Information System (NAHDIS) data summaries concerning abattoir condemnations due to WCS in sheep has shown an increase in the number of reported cases. Over the past 3 years, a total of 107 cases were reported in 2022, 547 in 2023, and 674 in 2024, highlighting the growing impact of this condition on the sheep industry (NAHDIS, 2024). In 2010, WCS-related losses were estimated at approximately R27 million (Webb and van Niekerk, 2011; Le Roux, 2012). The highest incidences of WCS have been recorded in the Northern Cape and Western Cape provinces, which account for 58% of the national sheep slaughter (https://www.rmis.co.za/statutory-statistics [Accessed on 18/05/2025]). Carcasses exhibiting WCS characteristics are rejected at abattoirs and are deemed unfit for human consumption due to the unappealing appearance and reduced shelf life (Simela, 2005; England et al., 2016). Consequently, WCS negatively impacts the sheep industry by causing financial losses. For instance, in 2018, 163 cases of WCS were reported among 143,000 sheep slaughtered at the KLK abattoir, resulting in losses of approximately R244 500 (du Pisanie, 2019). It is important to note that many abattoirs do not provide precise statistics on WCS cases, and the resulting decline in clientele further reduces income generation. Although affected animals appear physically normal before slaughter, WCS manifests after skin removal during processing, revealing an accumulation of watery fluid on the dorsal parts of the carcass, hind legs, and flanks (Brock et al., 1983; Hattingh et al., 1983) (Figure 1). These carcasses remain wet even after overnight cooling (Hattingh et al., 1983). However, a recent study by Hatting (2023) has demonstrated that meat from WCS-affected sheep can be processed into pet food and human-consumable products such as mince, sausages and cold meats.

Despite extensive research, the underlying causes of WCS remain elusive. The condition appears sporadically across different regions and production systems, making it difficult to predict or control. Several hypotheses have been proposed, including genetic predisposition, pre-slaughter handling, water retention issues, electrolyte imbalances and environmental factors (Brock et al., 1983; Joubert et al., 1985; Jansen and Pretorius, 1986). Stress during the pre-slaughter period has been suggested as a contributing factor, including the possibilities of over-hydration upon arrival at abattoirs, transport distances, allergic reactions and compulsory dipping (De La Fuente et al., 2010; Birhanu, 2020). Additional potential factors include high-pressure carcass washing, condensation in coolers and feed block provision before slaughter (Brock et al., 1983; Joubert et al., 1985). However, physiological responses to ante-mortem stress, such as dehydration, electrolyte imbalances, and glycogen depletion, have not been definitively linked to WCS (Schaefer et al., 2001). As a result, WCS remains a persistent challenge for researchers and livestock producers. Initially, WCS was hypothesized to be breed-specific, primarily affecting Dorper sheep and Dorper-crosses. This assumption may stem from the predominance of the Dorper breed in the Northern Cape, where WCS cases are most frequently reported (Joubert et al., 1985; Van der Westhuizen et al., 2019). However, recent abattoir reports indicate that WCS has also been observed in other commercial breeds, including SA Merino, SA Mutton Merino and Meatmaster (Van der Westhuizen et al., 2019). This raises the possibility that WCS is not confined to a single breed but may be influenced by a combination of genetic, environmental, and management-related factors. Consequently, further research is necessary to investigate the role of breed composition in WCS occurrence.

Previous sheep genomic studies primarily utilized microsatellite markers to assess population variation in South African commercial and indigenous breeds (Soma et al., 2012; Maqhashu et al., 2020). Advances in genomic technologies now allow for comprehensive genomic analysis using single nucleotide polymorphisms (SNPs). The decreasing cost of genomic technology has expanded research possibilities in various areas, including biodiversity assessment (Kijas et al., 2009), animal production (Rupp et al., 2016), disease susceptibility (Heaton et al., 2012), population structure (Dzomba et al., 2021), and the identification of genetic factors underlying phenotypic traits (Cargill et al., 2008). To date, only one relatively small genetic study has investigated the cause of WCS in sheep (Van der Westhuizen et al., 2019). The identification of two genetic markers associated with WCS suggests that genetics may contribute to its incidence in South African sheep (Van der Westhuizen, 2018).

Given the increasing evidence of WCS affecting multiple breeds, this study aims to assess whether breed composition plays a role in its occurrence. Recent reports indicate that abattoirs in multiple provinces have observed WCS in various sheep breeds. Breed composition, defined as the partition of an individual’s genome based on ancestry, has several practical applications (Wu et al., 2020). For example, in crossbred animals, understanding breed composition can help optimize breeding strategies by maximizing non-additive genetic effects (Mishra et al., 2017). The primary objective of this study is to investigate the breed composition of WCS-affected and unaffected sheep to determine whether genetic factors contribute to the condition. Since indigenous breeds are not widely used in commercial production systems due to their smaller body size and slower growth rates (Ngcobo et al., 2022), only commercial meat breeds were included in this study. The specific objectives were to (i) collect meat samples from WCS-affected and unaffected animals, (ii) extract DNA from these samples and genotype each animal using high-density SNP assays, (iii) use bioinformatics tools to assign individuals to specific breeds, and (iv) perform breed composition analysis to evaluate whether WCS incidence is linked to breed composition. This study hypothesizes that WCS is not confined to a single breed and that it may be detected in other breeds. Understanding the breed relationships of affected animals will provide critical insights into whether WCS has a genetic basis or if production systems and environmental conditions play a more significant role. This knowledge will ultimately aid in developing targeted interventions and improved management practices to mitigate economic losses associated with WCS.

## 2 Materials and methods

### 2.1 Tissue collection

Ethical approval was obtained at the ARC Animal Production Ethics Committee (APIEC23/07). The carcasses were classified as WCS-affected or unaffected based on abattoir grading and condemnation by the meat inspector, 24 h post slaughter at the abattoir. Also, both case and control animals were sampled from the same cohort to ensure consistency and comparability. Due to the sporadic nature of the condition, it is challenging to target specific breeds for sampling. Meat samples were collected from WCS-affected and unaffected carcasses at two commercial abattoirs: KLK Upington Abattoir (Northern Cape) and Cavalier Abattoir (Gauteng). Strict standard operating protocols were executed to ensure there was no cross contamination between tissue samples and between carcasses. The samples were taken from the hind leg (*biceps femoris* muscle) of the carcasses. Each sample was placed in an individual tube and stored at −80°C until further analysis.

### 2.2 Animal information

**FIGURE 2:**
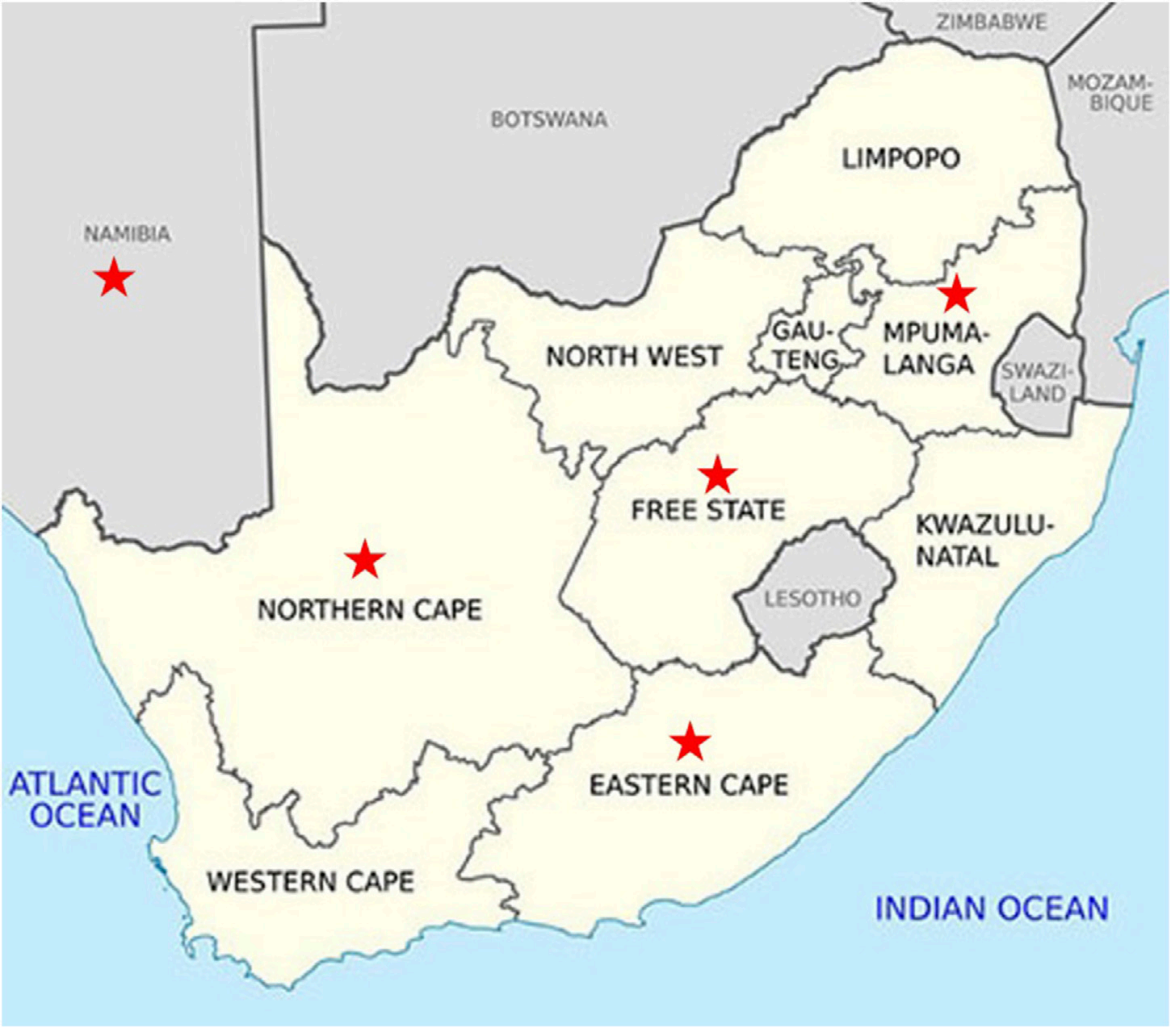
Map showing the regions (red star) from where the WCS-affected cases originated.

The study included WCS affected animals (n = 164, cases) and WCS unaffected animals (n = 83, controls) that originated from farms in the Northern Cape, Free State, Mpumalanga, Namibia, Eastern Cape and Gauteng (Figure 2). Namibia is situated north west of South Africa, bordering the Northern Cape province of South Africa. However, animals from Namibia are transported to the Northern Cape for processing at the abattoir. The sampled population comprised of Dorper, unknown Crossbreds, Meatmaster, Van Rooyen, White Dorper and Dorper x Meatmaster, SA mutton Merino and Merino sheep. All animals were under 1 year old (classified as A-grade lambs) and were graded A1 or A2 for carcass quality immediately after slaughter. Additional demographic factors, such as weight and production system (extensive vs. intensive), were recorded when available. In addition to the animals genotyped in this study, genotypes for reference animals were obtained from the Grootfontein Agricultural Development Institute (GADI) and Dzomba et al. (2021). The reference population contained representatives of Afrino (n = 51), Blackhead Persian (BHP; n = 14), Dohne Merino (n = 50), Dorper (n = 22), Meatmaster (n = 48), Merino (n = 91), Namakwa-Afrikaner (n = 10), South African Merino (SAM; n = 10), and South African Mutton Merino (SAMM; n = 10). Sheep genome Oar_v4.0 was selected as the reference genome for this study.

### 2.3 Ovine 50K SNP genotyping and quality control

For tissue lysis, 25 mg of meat sample was added to ATL buffer and Proteinase K, thereafter incubated at 56°C for 1 h. DNA was extracted from 200 μL of the resulting lysate using the Qiagen DNeasy Blood and Tissue extraction kit (Qiagen, Germany). Genomic DNA concentrations were measured using a Qubit^®^ 4.0 Fluorometer and standardized to 50 ng/μL (sample starting concentration between 10and 100 ng/μL). DNA samples were genotyped at the Agricultural Research Council - Biotechnology Platform, using the Ovine 50K SNP BeadChip (Illumina Inc., San Diego, CA, United States) that features 54,000 SNPs that are approximately equally distributed throughout the domestic sheep (*Ovis aries*) genome (Illumina, 2015; https://www.illumina.com). PLINK v1.9 (Purcell et al., 2007) was used for individual and SNP quality control (QC) for the genotyped and reference data. Quality control criteria included the following: SNP markers were excluded if minor allele frequency was less than 0.01 (--maf) and missing rate per SNP was greater than 0.05 threshold.

### 2.4 Breed composition analysis

To investigate the genome-wide relationships and divergences among the sheep populations, population structure analysis was conducted. Nei’s genetic distances were used to construct a neighbour-joining tree to visualize relationship between the study population and reference groups and plotted using R package “ggplot2” v3.5.1 in R v4.0.2. Fixation index (Fst) values between each pair of breeds with 95% level of confidence were calculated in R using the package “StAMPP” v 1.6.3 (Pembleton et al., 2013). Principal component analysis (PCA) was performed to describe continuous ancestral heterogeneity within the study population (Greenacre et al., 2022). The Eigen values were generated using the “pca” parameter in PLINK v 1.9, and a PCA plot generated using the packages “tidyverse” v 2.0.0 (Wickham et al., 2019) and “ggplot2” v3.5.1 in R v4.0.3 (R Core Team, 2023). Thereafter, a cluster analysis using the k-means method was performed using “cluster” v2.1.8.1 in R 4.5.0, where both the Elbow method and Silhouette analysis were used to determine the optimal K for the number of clusters for this study population.

To assess the relationships between province, WCS status, breed, and clustering patterns, statistical analyses were performed using chi-square tests, Cramér’s V, Fisher’s Exact Test, and multinomial logistic regression. Contingency tables were constructed for each categorical variable pair, and chi-square tests were conducted to determine whether observed distributions deviated significantly from expected values. The strength of associations was quantified using Cramér’s V, with values interpreted as weak (<0.2), moderate (0.2–0.4), or strong (>0.4) (Kearney and Cramer’s, 2017). Fisher’s Exact Test was employed as an alternative for cases with low expected counts, using 10,000 Monte Carlo simulations to ensure robust p-value estimation. Additionally, multinomial logistic regression models were fitted to evaluate the predictive relationships between province, WCS status and breed with clustering. Model fit was assessed using residual deviance and Akaike Information Criterion (AIC), with lower AIC values indicating better model performance. All statistical analyses were performed in R v 4.3.3 using the “stats” v 4.1.1 and “nnet” v 7.3–20 packages (Ripley et al., 2016) for chi-square tests and multinomial logistic regression, respectively. The “vcd” v1.4-13 package (Meyer et al., 2024) and ‘“rcompanion” v 2.5.0 (Mangiafico, 2025) was used for Cramér’s V calculations, and Fisher’s Exact Test was conducted using the ‘fisher.test’ function with simulated p-values.

Ancestry matrix analysis was employed to determine whether certain genetic admixtures are more prevalent in WCS-affected vs. unaffected animals. Individual ancestry coefficients were calculated using the sparse non-negative matrix factorization (sNMF) with the R package, LEA v 3.14.0 (Frichot and François, 2015) with K (i.e., number of hypothetical ancestors) values ranging from 1 to 12, with five iterations each. The optimal number of ancestors was set as the lowest cross-entropy value. The resulting Q values (the ancestry coefficients) were plotted breed-wise to show proportion of ancestry from different breeds in each individual animal (Patel et al., 2022) using the ‘plotQMultiline’ function in “Pophelper” v2.3.1 package (Francis, 2017) in R v 4.1.1. The K value is the number of sub-populations that make up the total population, and is determined by the total number of reference populations that is included in the analysis (Alexander et al., 2015).

## 3 Results

### 3.1 Animal information

**FIGURE 3:**
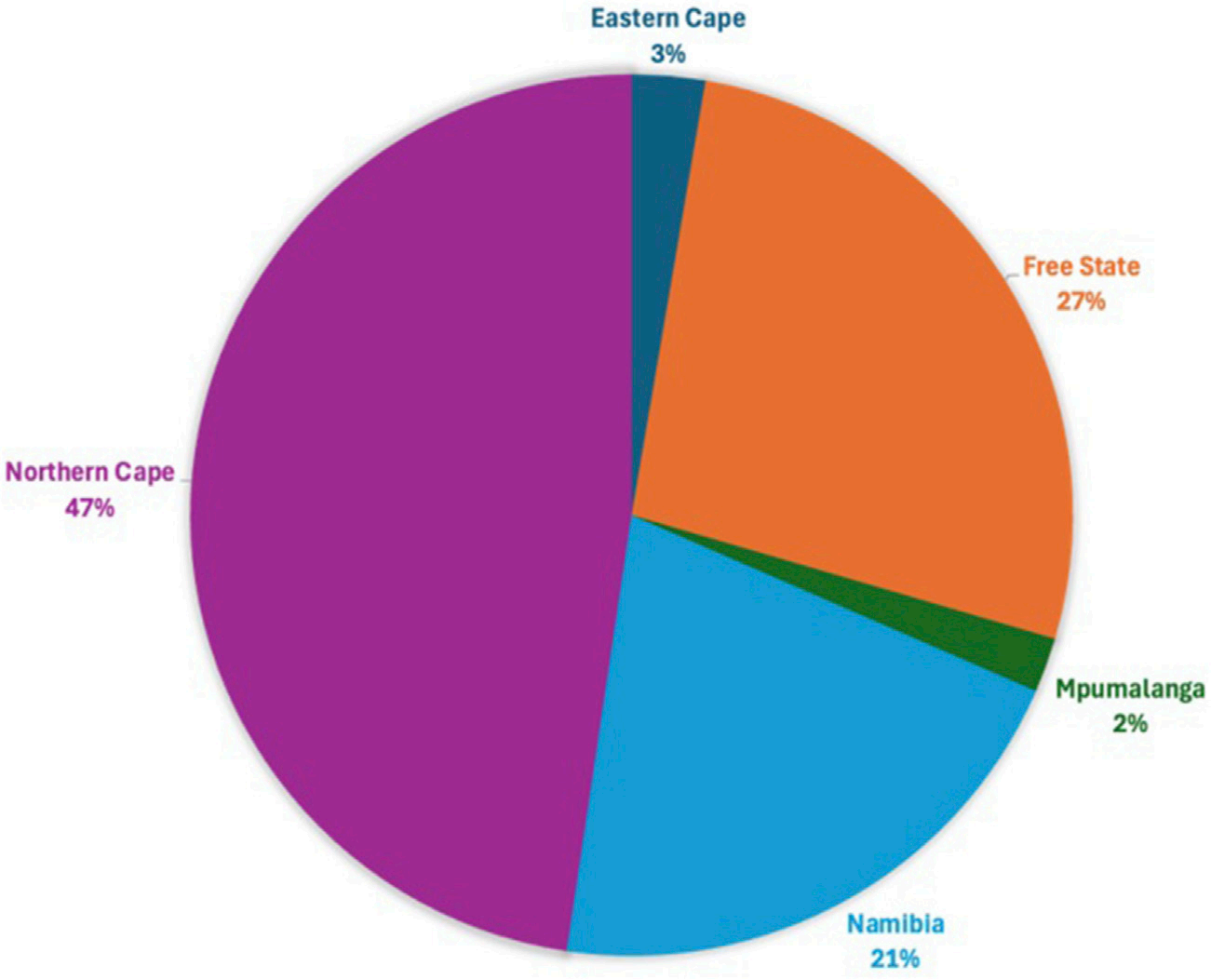
The proportion of WCS-affected cases from the various regions.

**FIGURE 4:**
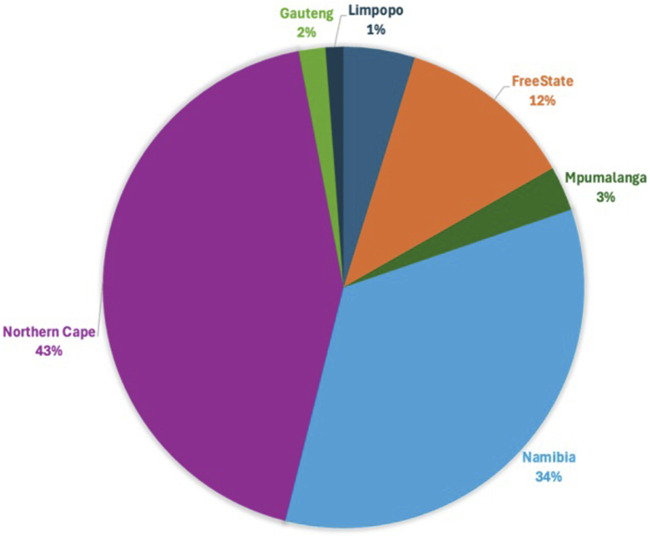
The proportion of WCS-unaffected cases from the various regions.

Following quality control, 34,528 SNPs and 540 animals remained. This comprised 164 affected animals and 376 unaffected animals which included the reference populations. The majority of affected were collected in the Northern Cape (47%), Free State (19%) and Namibia (21%), while the majority of unaffected were collected in the Northern Cape (43%) and Namibia (34%) (Figures 3, 4). The majority of affected animals of which the breed status was known, were Dorper (73%) and Merino (19%) sheep. The remaining breeds (8%), included Dorper x Meatmaster crossbred, Meatmaster, unknown Crossbred, White Dorper and Van Rooyen. Among the unaffected animals of which the breed status was known, included Merino (30%), followed by Dorper (15%), Afrino (14%), Dohne Merino (14%), Meatmaster (13%). The remaining breeds 29%, included SA Merino, Namakwa-Afrikaner, Crossbred, Blackhead Persian, as well as SA Mutton Merino sheep.

### 3.2 Breed composition analysis

**FIGURE 5:**
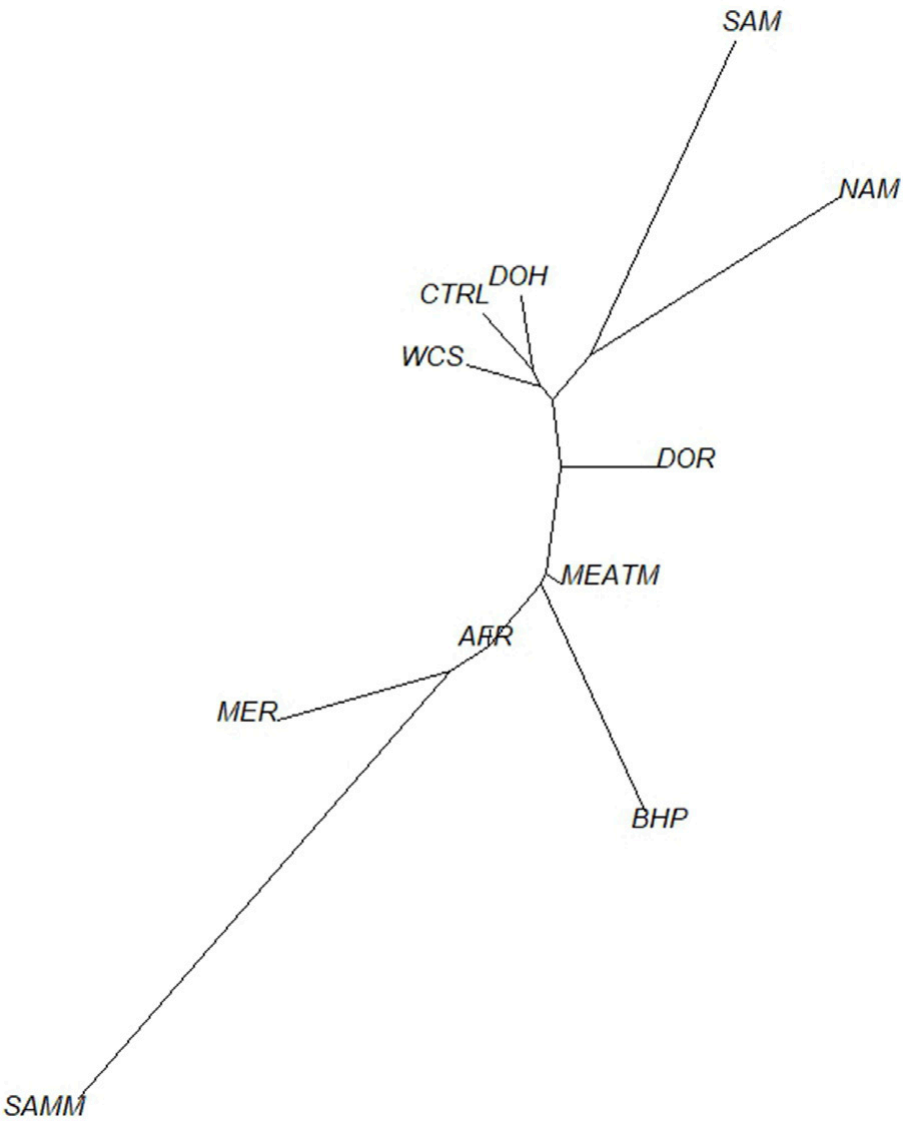
Neighbor-joining tree of WCS-affected and reference sheep population based on Nei’s genetic distances. Breeds included in the analysis were Afrino (AFR), Blackhead Persian (BHP), unaffected animals (CTRL), Dohne Merino (DOH), Dorper (DOR), Meatmaster (MEATM), Merino (MER), Namakwa-Afrikaner (NAM), South African Merino (SAM), South African Mutton Merino (SAMM) and Wet carcass syndrome-affected (WCS).

**FIGURE 6:**
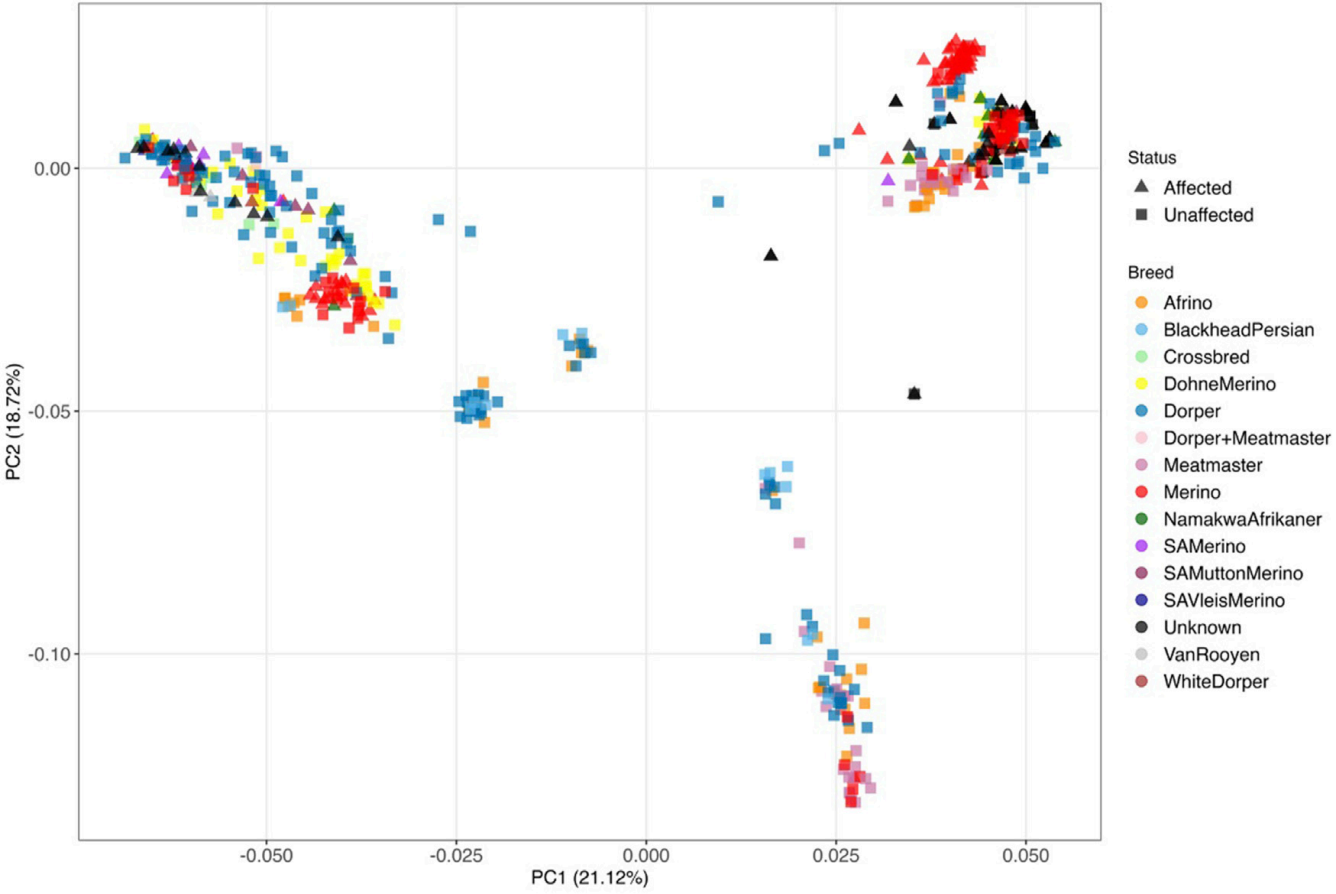
Principal component analysis of WCS-affected (indicated with triangles) and WCS-unaffected (indicated with blocks) sheep for the different breeds with PC1 vs. PC2.

**FIGURE 7:**
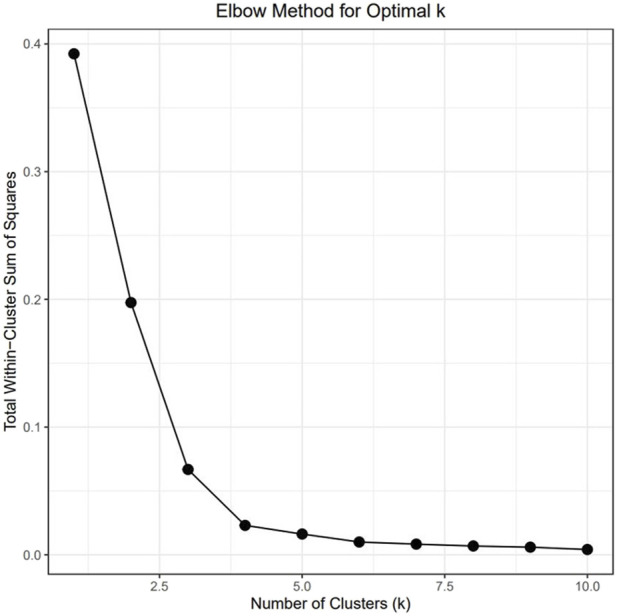
The Elbow method for optimal K clusters present.

**FIGURE 8:**
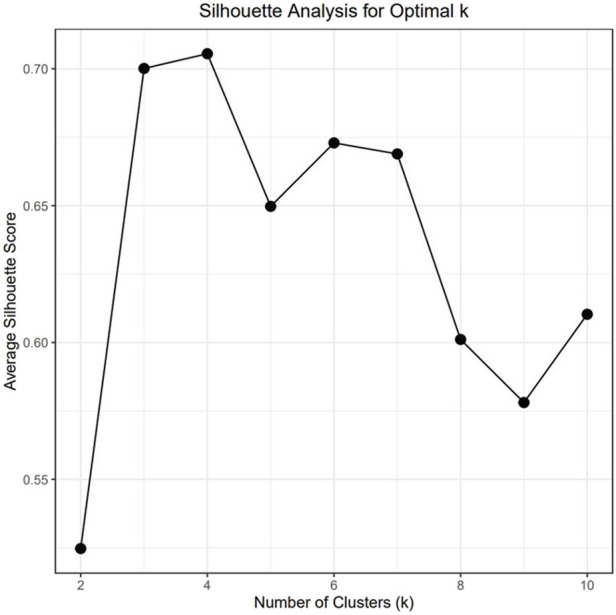
Average silhouette score for validation of clusters present.

**FIGURE 9:**
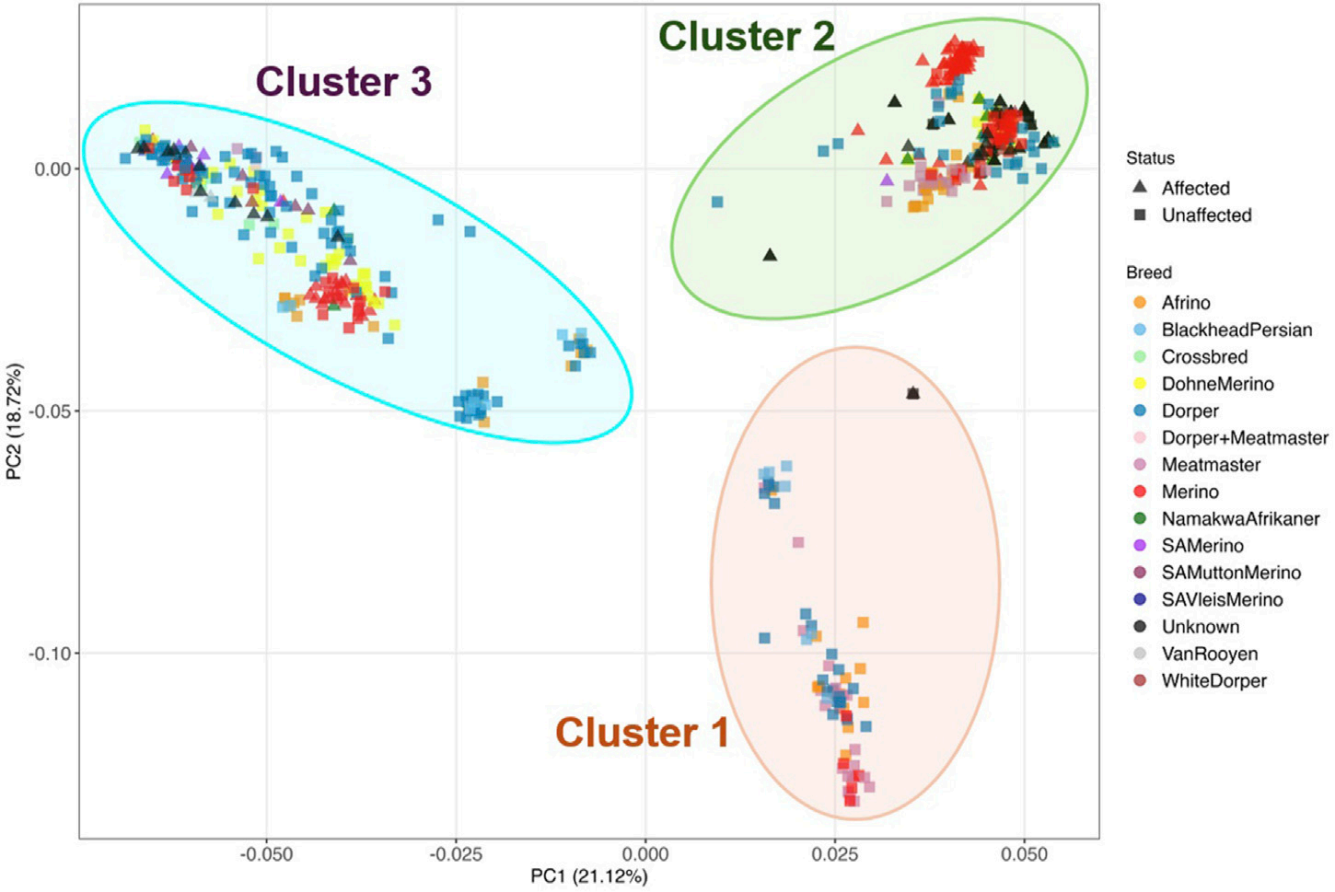
Based on the k-means clustering the population were separated into three clusters labelled 1-3.

**FIGURE 10:**
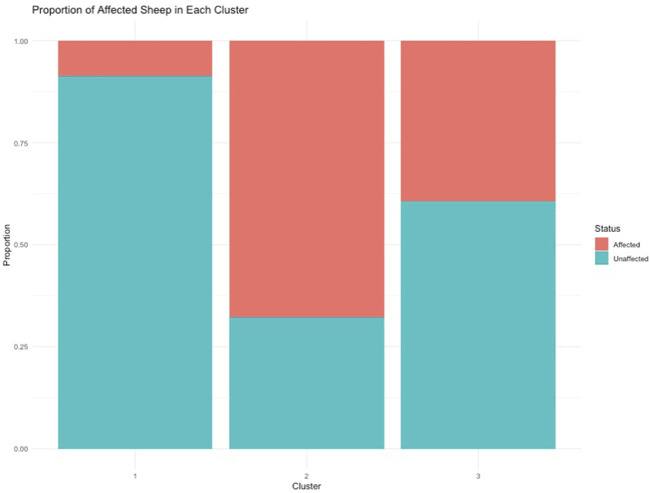
Proportion of WCS-Affected and unaffected sheep in each cluster.

**FIGURE 11:**
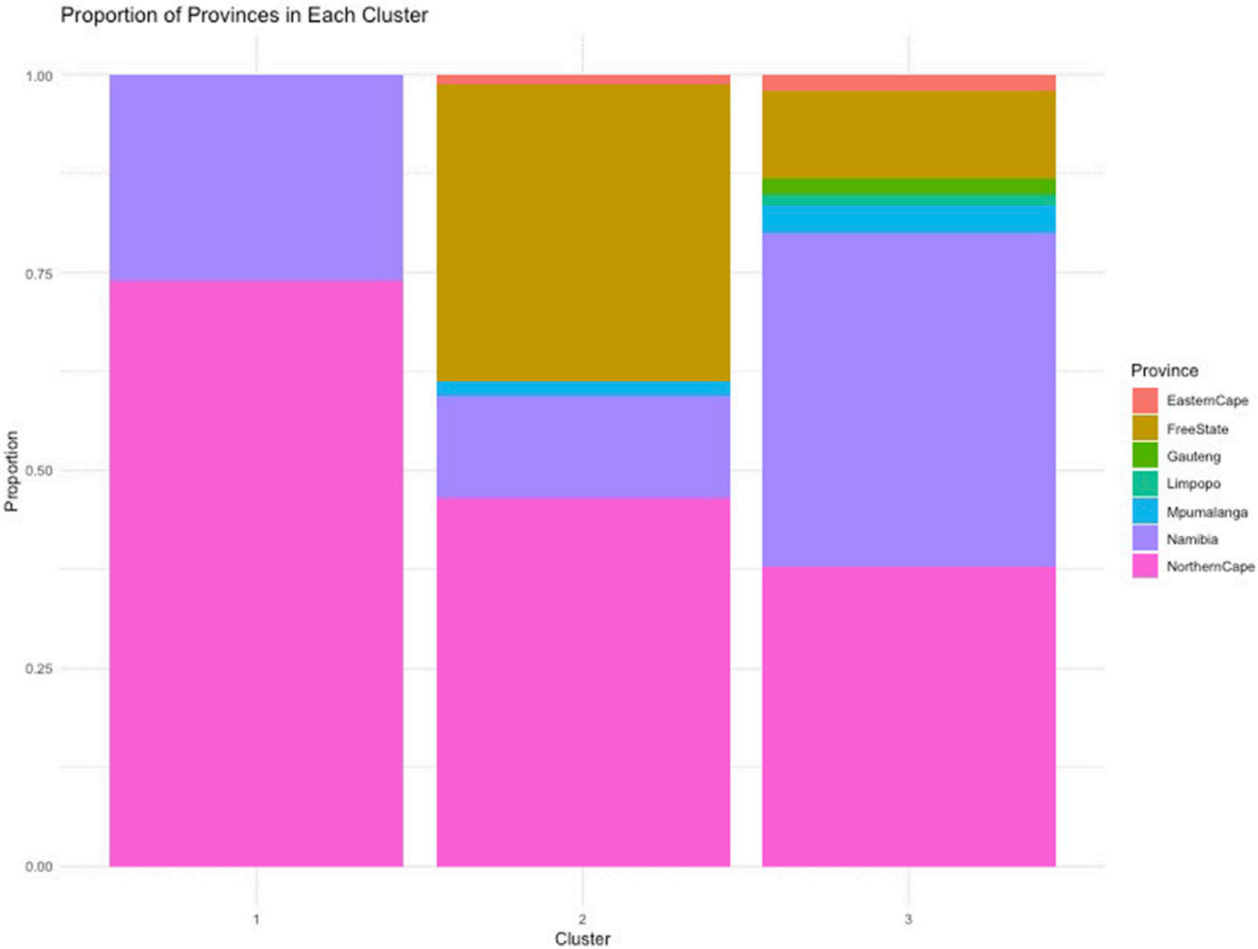
Proportion of sheep from each province that were observed in each cluster.

To understand the relationship between the WCS affected and unaffected populations in relation to reference populations, a neighbor-joining tree was constructed (Figure 5). The WCS affected and unaffected populations are grouped closely together. Furthermore, it shows the Dohne Merino and Dorper populations are close to the WCS-affected and unaffected populations. Figure 6 shows the results of PCA analyses that maximally explain the variance of all the variables across all SNP loci. The first (21.12%) and second (18.72%) principal components accounted for 39.84% of the variability. PCA analyses revealed 3 major clusters which was validated using k-means clustering analysis. Furthermore, the elbow method (Figure 7) helps determine the optimal number of clusters (K) by plotting within-cluster sum of squares (WCSS) against the number of clusters, while the average silhouette score (Figure 8) validates the quality of the clusters, with higher scores indicating better separation (Dalmaijer et al., 2022). The point where the curve starts to flatten out (the “elbow”) suggests the optimal K which is K = 3 in the study population (Figure 7). In addition, an average silhouette score of 0.7–1.0 indicates strong evidence for clustering and the location of the maximum is considered as the appropriate number of clusters, which is K = 3 in this study population (Figure 8). The PCA also showed the affected samples to reside in two of the clusters, supporting the interpretation that the origin of WCS is not breed-specific (Figure 9). With the exception of 2 samples, the affected and unaffected populations grouped in Clusters 1–3 (Figure 10). Since the affected animals were observed to reside in two major Clusters (2 and 3), suggested that additional factors beyond genetic background contribute to WCS occurrence. Even though the affected and unaffected populations grouped in Clusters 1, 2 and 3, the majority of affected animals were located in Cluster 2, while the majority of unaffected populations were located in Cluster 1. While the Merino were present in all 3 clusters, Dorper was only present in 2 clusters. It is interesting to note that the majority of affected Merino were observed in Cluster 1, while the unaffected Merino resided in Clusters 1 and 3. Majority of affected Dorper grouped in Cluster 3. Geographical location of the animals in each cluster reveal that each cluster has the largest proportion of animals from the Northern Cape province, with the highest present in cluster 1 (Figure 11). The second largest proportion for cluster 1 and 3 animals originating from Namibia while cluster 2 has animals from the Free State province. These clusters may reflect different production environments, regional variations, or management practices at the sampled abattoirs.

**FIGURE 12:**
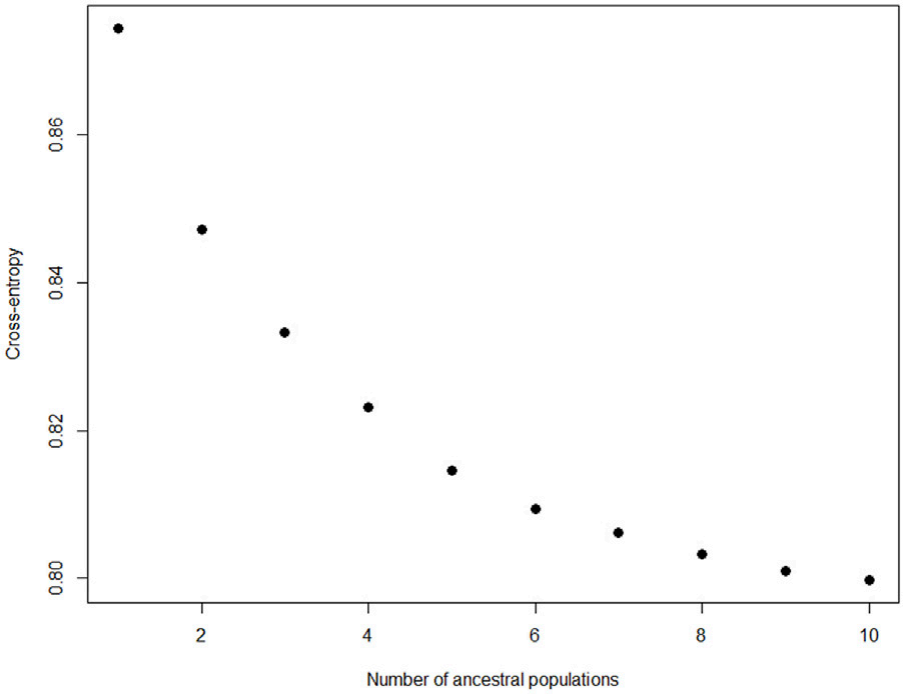
Cross-entropy values to determine the number of ancestral population for ancestry matrix analysis.

**FIGURE 13:**
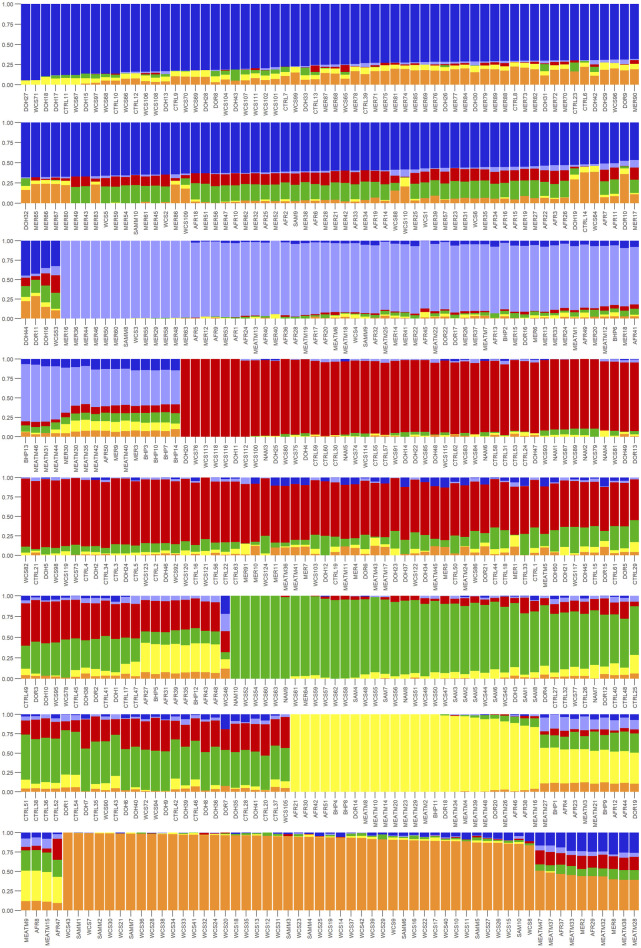
Ancestry matrix of WCS-affected sheep at K = 6 on an individual level. The y-axis showing the ancestry probability values and the x-axis shows each individual.

Ancestry-based clustering uncovered the diversity and breed relations of the populations under study. The cross-entropy criterion assists in selecting the number of ancestral populations or a best run for a fixed value of K. A smaller value of cross-entropy means a better run in terms of prediction capability (Frichot and François, 2015). The cross-entropy criterion that is based on the prediction of masked genotypes to evaluate the fit of a model with K population indicated that the sheep in the present study descended from 6 populations (Figure 12). The population structure revealed unique genomic signatures in the WCS affected and unaffected populations with increasing K values. The ancestry at the population level is quite diverse for most animals of a particular breed, like the merino-types, hence clear admixture patterns are difficult to distinguish in a single breed. One possible reason could be due the origin ancestry of the Merino breed, as intensive gene flow, founder effects and geographic isolation are the main factors that determined the genetic makeup of current Merino and Merino-derived breeds (Ciani et al., 2015). Therefore, the admixture analysis was run per individual and then sorted by ancestry probabilities, placing individual WCS affected and unaffected samples next to a reference breed. K = 6 was selected as it corresponds to the inflection point of the cross-entropy curve. The value of K = 6 corresponds closely to geographical distributions within the sample, that align with breeding history and population management practices in South African sheep. Thus, at the individual level of K = 6, the ancestry matrix shows the clustering of both affected and unaffected individuals with the individuals of the various reference breeds (Figure 13; Supplementary Table S1). The LEA program automatically assigned color codes to each individual based on K-value and ancestry. Most WCS individuals were clustered with the Merino-type and Dorper animals confirming the results observed in the neighbor joining unrooted tree (Figure 5). Also, the WCS affected displayed admixed signatures from all the Merino-type populations. The lack of a single ancestral population being significantly represented in an overwhelming majority of affected animals is viewed as evidence that WCS does not originate from one particular breed.

Population pairwise FST ranged from 0.008 (between the WCS affected sheep and Dohne Merino) to 0.036 (between WCS affected and Blackhead Persian sheep populations) (Table 1). This observation is also seen in Figure 4, where the WCS-affected population is genetically closer to the Dohne Merino than the Blackhead Persian population. The neighbour-joining tree (Figure 5) illustrates genetic differentiation among the populations that were studied. In contrast, estimates of FST account for genetic sampling and reflect differences in the allele frequency distributions. While Nei’s distance quantifies the overall genetic difference between populations and is often used to estimate divergence time. As a result, FST and Nei’s genetic distance measure different properties of the populations under study (Holsinger and Weir, 2009).

**TABLE 1:** Mean pair-wise fixation index (F_ST_) values with confidence intervals in brackets at a 95% level among WCS-affected, unaffected and reference populations.

	AFR	BHP	DOH	DOR	MEATM	MER	NAM	SAM	SAMM	WCS
BHP	0.013 (0.012–0.013)									
DOH	0.026 (0.026–0.027)	0.041 (0.040–0.042)								
DOR	0.010 (0.010–0.011)	0.015 (0.014–0.015)	0.008 (0.007–0.008)							
MEATM	0.008 (0.007–0.008)	0.004 (0.003–0.004)	0.027 (0.026–0.027)	0.002 (0.001–0.002)						
MER	0.014 (0.013–0.014)	0.034 (0.033–0.034)	0.042 (0.041–0.042)	0.039 (0.038–0.040)	0.024 (0.023–0.024)					
NAM	0.041 (0.040–0.042)	0.050 (0.048–0.051)	0.016 (0.016–0.017)	0.019 (0.018–0.020)	0.029 (0.028–0.030)	0.082 (0.080–0.083)				
SAM	0.027 (0.026–0.028)	0.036 (0.034–0.037)	0.028 (0.027–0.029)	0.017 (0.016–0.018)	0.025 (0.024–0.026)	0.070 (0.069–0.071)	0.021 (0.020–0.023)			
SAMM	0.032 (0.031–0.033)	0.054 (0.052–0.055)	0.070 (0.069–0.070)	0.064 (0.062–0.065)	0.038 (0.037–0.039)	0.047 (0.046–0.048)	0.113 (0.112–0.115)	0.088 (0.086–0.089)		
WCS	0.022 (0.021–0.022)	0.036 (0.035–0.037)	0.008 (0.007–0.008)	0.010 (0.009–0.010)	0.022 (0.022–0.023)	0.033 (0.032–0.033)	0.025 (0.025–0.026)	0.021 (0.020–0.022)	0.029 (0.029–0.030)	
UAFF	0.029 (0.029–0.030)	0.040 (0.039–0.041)	0.004 (0.004–0.005)	0.008 (0.007–0.008)	0.026 (0.025–0.027)	0.052 (0.051–0.052)	0.012 (0.011–0.013)	0.026 (0.025–0.026)	0.079 (0.078–0.081)	0.011 (0.010–0.011)

Afrino (AFR), Blackhead Persian (BHP), unaffected animals (UAFF), Dohne Merino (DOH), Dorper (DOR), Meatmaster (MEATM), Merino (MER), Namakwa-Afrikaner (NAM), South African Merino (SAM), South African Mutton Merino (SAMM) and Wet carcass syndrome-affected (WCS).

The statistical analysis revealed significant associations between province, status, and breed with clustering patterns (Tables 2 and 3). The chi-square tests indicated highly significant relationships across all comparisons (p < 0.0001), confirming that the observed distributions were not random (Table 2). The strength of these associations varied, with Cramér’s V values ranging from 0.35 to 0.36 based on province while, for breed it ranges from 0.25 to 0.60. Notably, breed exhibited the strongest association with clustering (Cramér’s V = 0.36), followed by WCS status (Cramér’s V = 0.25), suggesting that both genetic and management-related factors influence clustering (Table 3). Province also demonstrated a moderate effect (Cramér’s V = 0.32), indicating a geographic component in the clustering patterns (Table 2). The relationship between WCS status and breed was particularly pronounced, with a chi-square statistic of 166.92 (df = 11, p < 2.2e-16) and a strong effect size (Cramér’s V = 0.60), suggesting that breed composition varies significantly between the WCS affected group and the unaffected group (Table 2). Similarly, breed was significantly associated with clustering (X^2^ = 126.79, df = 22, p < 2.2e-16), reinforcing the idea that genetic or phenotypic factors contribute to cluster formation. The Fisher’s Exact Test, performed with 10,000 simulations, consistently confirmed these associations, providing additional robustness to the statistical findings (Tables 2 and 3). Multinomial logistic regression models further supported these associations, with relatively low AIC values across comparisons, indicating a good model fit. The model for breed vs. cluster showed the lowest AIC (327.37), followed by WCS status vs. province (428.90), suggesting that these variables play a key role in defining clustering patterns. Collectively, these results highlight the significant influence of both geographic (Province) and biological (WCS status and breed) factors in shaping the observed clusters (Tables 2 and 3).

**TABLE 2:** Statistical tests based per province.

Test		Province vs. cluster	WCS status vs. cluster	WCS status vs. cluster
Chi-squared	Value	66.22	41.77	30.04
	Degree of freedom (df)	12	2	6
	*P-value*	1.619e-09	8.498e-10	3.871e-05
Cramér’s V	Value	0.32	0.36	0.31
Fisher’s Exact Test	*P-value*	9.999e-05	9.999e-05	9.999e-05
Multinomial Logistic Regression	Residual Deviance	507.12	536.57	414.90
	AIC	535.12	544.57	428.90

**TABLE 3:** Statistical tests based per breed.

Test		Breed vs. cluster	Breed vs. cluster	WCS status vs. cluster
Chi-squared	Value	126.79	166.92	27.68
	Degree of freedom (df)	22	11	2
	*P-value*	2.2e-16	2.2e-16	9.756e-07
Cramér’s V	Value	0.36	0.60	0.25
Fisher’s Exact Test	*P-value*	9.999e-05	9.999e-05	9.999e-05
Multinomial Logistic Regression	Residual Deviance	790.26	303.37	905.51
	AIC	838.26	327.37	913.51

## 4 Discussion

This is the first study to estimate the breed composition of South African sheep challenged with wet carcass syndrome. The data showed breed-specific genetic stratification. Nel et al. (2022) also observed South African and Australian Dorper’s clustering together, along with Dohne Merino and South African Mutton Merino. In addition, breed exhibited the strongest association with cluster assignment (χ^2^ = 196.79, p < 2.7e-16; Cramér’s V = 0.36), and breeds such as Dorper (72%) showed breed-specific genetic stratification independent of sampling location. These findings support the hypothesis that conserved genetic lineages dominate population structure in these breeds.

A genetic predisposition to WCS is indicated by the strong association of breed and health status (χ^2^ = 166.97, p < 2.7e-16; Cramér’s V = 0.60), and multinomial regression confirmed breed as the strongest predictor of both cluster membership (AIC = 838.26) and health status (AIC = 327.37). These findings imply that breeding strategies prioritizing traits like meat yield or adaptability may inadvertently increase genetic vulnerabilities to WCS, particularly in Dorper-dominated regions such as the Northern Cape. Genetic factors likely contribute to WCS susceptibility, as supported by the identification of genomic regions associated with the condition (Van der Westhuizen et al., 2019). Studies have reported a higher incidence of WCS in Dorper crosses compared to other breeds, reinforcing the role of genetic background (Brock et al., 1983; Hattingh et al., 1983; Van der Westhuizen et al., 2019). Wet Carcass Syndrome appears to be a complex, multifactorial condition influenced by both genetic predisposition and environmental or management-related factors. In particular, transport stress has been shown to increase plasma concentrations of cortisol, adrenaline, nor-adrenaline and dopamine, increased weight loss and compromise meat quality (Kadim et al., 2006). Hydration status, ambient temperature, and handling methods are known to modulate physiological stress responses that contribute to fluid retention and altered carcass quality (Ferguson and Warner, 2008). The study by Joubert et al. (1985) had tried to induce the WCS condition by water deprivation and subsequent over hydration, results showed that the intake of feed with high salt content can exacerbate the condition. Although genetic ancestry primarily determined cluster formation, province (Cramér’s V = 0.36) and environmental factors also contributed to population heterogeneity. The Northern Cape abattoir, sourcing animals from a predominantly Dorper/Merino production region, exhibited clusters reflecting breed uniformity. This may be attributed to the highest number of sheep farms that located in the Eastern Cape province, followed by Northern Cape and Free State provinces (Retief, 2020). In contrast, the Gauteng abattoir, which draws sheep from diverse provinces and management systems, produced genetically mixed clusters. The weaker association between health status and cluster membership (Cramér’s V = 0.25) suggests that environmental stressors, such as feed quality, climate, or farm-specific practices, may influence WCS risk in genetically predisposed breeds. The interaction between breed-specific traits and environmental factors may further contribute to WCS development. Ferreira et al. (2015) noted that different sheep genotypes exhibit varying responses to dietary changes, which could affect carcass quality and predispose certain breeds to conditions like WCS. The study by Brand (2000) showed that the Dorper were less-selective grazers than the Merino and used a larger range of different plant species than the Merino. This is particularly relevant for Dorper sheep, whose rapid growth and high carcass yield may make them more susceptible to metabolic disorders under specific management practices (Zhao et al., 2016; Saeed et al., 2018).

The results suggest that environmental and management practices may influence WCS incidence. The genetic clusters observed in this study likely reflect regional breeding preferences. The Eastern Cape cluster aligns with regional selection for hardier, drought-resistant breeds. Similarly, genetic isolation in certain breeds, such as the Dorper, suggests targeted selection for traits like rapid growth and fat deposition. Merino sheep have been less frequently associated with WCS than Dorper, although differences in fat distribution and muscle yield between Dorper and Merino breeds suggest varying physiological responses to environmental and nutritional factors (Burger et al., 2014; Brand et al., 2018). Suliman et al. (2021) found that different breeds’ exhibit varying fattening performances and carcass characteristics, while Dorper sheep may be more prone to WCS due to their rapid growth and high carcass yield, environmental and management factors also play a role. Further research is required to fully elucidate the genetic and environmental interactions contributing to WCS in these breeds. However, such selection strategies may prioritize short-term productivity at the expense of long-term health risks, as evidenced by the strong breed-status associations observed in this study. Farmers and researchers should balance production goals with genomic diversity to mitigate WCS incidence. In addition, population pairwise F_ST_ analysis supported population clustering by reporting high genetic distance between WCS affected population and Blackhead Persian while low genetic distance with Dohne Merino and Dorper populations. This observation was also evident in the neighbour-joining tree but with Meatmaster having a closer relationship to WCS affected than the South African Mutton Merino. In this study, the Blackhead Persian was highly differentiated from the WCS affected population with F_ST_ of 0.036 while the Dorper was the least differentiated from the WCS affected population. A similar trend was observed by Dzomba et al. (2020) which could be attributed to the breed differences, where the Blackhead Persian is a fat tailed and hair type breed while the Dohne Merino is a wool-type sheep used for meat production.

Overall, WCS is strongly associated with breed, particularly Dorper and Merino-types showing heightened susceptibility due to genetic and physiological traits. However, environmental and management factors, such as regional farming practices and dietary conditions, may also contribute to its expression. This observation aligns with the gene-environment interaction model in complex diseases, where genetic predisposition confers underlying susceptibility and environmental exposure acts as triggers that influence development of WCS.

## 5 Conclusion

This is the first study to determine the breed composition of South African sheep affected by WCS. The results provide valuable information on the population structure of WCS affected sheep in South Africa. Overall, indicating that WCS is not limited to a specific breed, and is prevalent in various crossbred sheep as well. To mitigate WCS, future efforts should integrate genomic studies to identify risk loci alongside tailored management strategies addressing both genetic and environmental contributors. Such an approach would support sustainable breeding programs and enhance flock resilience across diverse production systems.

## Data Availability

The datasets presented in this article are not readily available because they are the property of the S.A sheep producers, and the information in these datasets is commercially sensitive. Requests to access the datasets should be directed to the corresponding author.
