# Supplementary material for: Estimation of breed composition of South African sheep affected with wet carcass syndrome
Source: Front Genet. 2025 Jul 21;16:1635947. doi: 10.3389/fgene.2025.1635947 (PMC12318712; doi:10.3389/fgene.2025.1635947)
Supplement: Supplementary file 1 [file DataSheet1.pdf]

Supplementary Material

1 Supplementary Data

Supplementary Table 1: Q-values for the WCS affected and unaffected animals with the reference populations.

Animal ID	Breed	K = 6					
DOH27	DohneMerino	0.944678	1.00E-04	1.00E-04	1.00E-04	0.054922	1.00E-04
WCS71	WCS-affected	0.941712	1.00E-04	1.00E-04	1.00E-04	0.057888	1.00E-04
DOH18	DohneMerino	0.896759	1.00E-04	1.00E-04	0.004517	0.098423	1.00E-04
DOH17	DohneMerino	0.881154	0.002735	1.00E-04	0.037407	0.078504	1.00E-04
CTRL11	Unaffected	0.879235	1.00E-04	1.00E-04	0.036542	0.082044	0.001979
WCS67	WCS-affected	0.877216	1.00E-04	1.00E-04	0.040047	0.082438	1.00E-04
DOH15	DohneMerino	0.863338	1.00E-04	1.00E-04	0.056291	0.066943	0.013227
WCS97	WCS-affected	0.862276	0.003933	0.002785	0.054808	0.051768	0.02443
WCS68	WCS-affected	0.861888	1.00E-04	0.002643	0.039341	0.045591	0.050437
CTRL10	Unaffected	0.860829	1.00E-04	0.012992	0.039675	0.036536	0.049867
WCS66	WCS-affected	0.840815	0.019481	1.00E-04	1.00E-04	0.063838	0.075666
CTRL12	Unaffected	0.839832	0.017616	1.00E-04	1.00E-04	0.063169	0.079183
WCS106	WCS-affected	0.837103	0.017708	0.017484	0.011701	0.07158	0.044423
WCS108	WCS-affected	0.83407	0.018867	0.018496	0.039512	0.053526	0.03553
DOH13	DohneMerino	0.833269	0.033992	1.00E-04	0.033195	0.059989	0.039455
CTRL9	Unaffected	0.826333	0.00261	1.00E-04	0.001926	0.064212	0.104819
WCS70	WCS-affected	0.825105	0.003973	1.00E-04	0.0051	0.065584	0.100138
WCS69	WCS-affected	0.820297	1.00E-04	1.00E-04	1.00E-04	0.083313	0.09609
DOH28	DohneMerino	0.818345	1.00E-04	1.00E-04	1.00E-04	0.083687	0.097669
DOR8	Dorper	0.814599	0.014994	1.00E-04	0.065738	0.072659	0.03191
WCS104	WCS-affected	0.812171	0.004336	0.007967	0.051621	0.058766	0.06514
DOH43	DohneMerino	0.811156	0.000449	1.00E-04	0.135804	1.00E-04	0.052391
WCS107	WCS-affected	0.800635	0.010382	0.002878	0.056791	0.073421	0.055893
WCS111	WCS-affected	0.79473	1.00E-04	0.023765	0.038612	0.061031	0.081763
WCS102	WCS-affected	0.793615	0.026049	0.001036	0.066184	0.054436	0.05868
WCS101	WCS-affected	0.782085	0.029169	1.00E-04	0.050062	0.066498	0.072087
CTRL7	Unaffected	0.7761	1.00E-04	0.003119	0.061833	0.049676	0.109172
WCS99	WCS-affected	0.770372	0.008991	1.00E-04	1.00E-04	0.067623	0.152814
DOH33	DohneMerino	0.767287	0.019027	0.001839	0.094895	0.054596	0.062357
CTRL13	Unaffected	0.756449	0.00647	0.080596	0.035373	0.054114	0.066997
MER87	Merino	0.750593	0.029511	0.011116	0.00574	0.056969	0.146072
MER68	Merino	0.748676	0.029375	0.021474	1.00E-04	0.061107	0.139268
WCS65	WCS-affected	0.748013	0.005417	0.088807	0.032279	0.050568	0.074917
MER78	Merino	0.741629	0.029169	0.003801	0.00113	0.057395	0.166875

CTRL39	Unaffected	0.732818	0.04783	0.003645	1.00E-04	0.084283	0.131324
MER71	Merino	0.729022	0.03084	0.01836	0.004148	0.051217	0.166413
MER75	Merino	0.727172	1.00E-04	0.030175	1.00E-04	0.069823	0.172631
MER81	Merino	0.726262	0.001769	0.024251	1.00E-04	0.049945	0.197675
MER74	Merino	0.726188	0.023636	0.011542	1.00E-04	0.043564	0.19497
MER85	Merino	0.722909	0.011242	0.021262	1.00E-04	0.064991	0.179496
MER69	Merino	0.718754	0.031983	0.001085	1.00E-04	0.066154	0.181923
MER76	Merino	0.716487	0.032495	0.013135	1.00E-04	0.06407	0.173712
DOH26	DohneMerino	0.713473	0.032114	0.013802	0.024008	0.045268	0.171335
MER77	Merino	0.713246	0.023661	0.024494	0.000231	0.047559	0.190808
MER84	Merino	0.711655	0.018564	0.021475	1.00E-04	0.046394	0.201813
DOH30	DohneMerino	0.710651	0.05006	1.00E-04	0.003628	0.079569	0.155992
MER79	Merino	0.710149	0.019366	0.031243	1.00E-04	0.044491	0.194651
MER89	Merino	0.707799	0.013034	0.030297	1.00E-04	0.05848	0.19029
MER88	Merino	0.704541	0.027904	0.040144	1.00E-04	0.052115	0.175197
CTRL8	Unaffected	0.704092	0.015419	0.001372	1.00E-04	0.053638	0.22538
MER73	Merino	0.702255	0.018351	0.008883	1.00E-04	0.058901	0.211509
MER82	Merino	0.700888	0.012418	0.028	1.00E-04	0.065687	0.192907
DOH31	DohneMerino	0.70047	0.034367	0.009598	0.087034	0.052695	0.115837
MER72	Merino	0.698802	0.020241	0.039308	1.00E-04	0.056389	0.185161
MER70	Merino	0.698682	0.017639	0.031199	1.00E-04	0.059007	0.193374
CTRL23	Unaffected	0.697298	1.00E-04	0.028093	0.110248	1.00E-04	0.164161
CTRL6	Unaffected	0.693672	0.009993	1.00E-04	0.020024	0.053886	0.222325
DOH42	DohneMerino	0.693294	0.054288	0.030392	0.063171	0.047686	0.111169
DOH29	DohneMerino	0.689979	0.035413	1.00E-04	1.00E-04	0.090995	0.183413
WCS96	WCS-affected	0.688668	0.042048	1.00E-04	1.00E-04	0.066763	0.202321
DOR9	Dorper	0.687872	0.058127	0.004571	1.00E-04	0.071294	0.178036
MER90	Merino	0.680995	0.015497	0.04433	1.00E-04	0.066	0.193079
DOH32	DohneMerino	0.680129	0.012429	1.00E-04	0.095704	0.048961	0.162677
MER65	Merino	0.678685	0.001557	0.041241	1.00E-04	0.041143	0.237275
MER66	Merino	0.677589	0.022685	0.011839	1.00E-04	0.049562	0.238226
MER67	Merino	0.674633	0.008387	0.029028	0.006652	0.045639	0.23566
MER80	Merino	0.674579	0.019738	0.030109	0.000378	0.053611	0.221584
MER49	Merino	0.670301	1.00E-04	0.129906	0.199493	1.00E-04	1.00E-04
MER43	Merino	0.669295	1.00E-04	0.120971	0.209434	1.00E-04	1.00E-04
MER83	Merino	0.666118	0.030574	0.035714	1.00E-04	0.038219	0.229276
WCS5	WCS-affected	0.660387	0.028819	0.103753	0.206842	1.00E-04	1.00E-04
MER59	Merino	0.656435	0.014546	0.126889	0.195897	0.006133	1.00E-04
MER54	Merino	0.656102	0.019154	0.103858	0.220686	1.00E-04	1.00E-04
SAMM10	SAMuttonMerino	0.650206	0.004259	0.122939	0.222397	1.00E-04	1.00E-04
MER61	Merino	0.647737	0.021308	0.118721	0.195933	0.000802	0.015499
MER45	Merino	0.646499	0.01274	0.125022	0.215539	1.00E-04	1.00E-04
WCS2	WCS-affected	0.646067	0.000708	0.146591	0.194837	1.00E-04	0.011696
MER86	Merino	0.643903	0.045299	0.03425	1.00E-04	0.046765	0.229683

WCS109	WCS-affected	0.632034	0.020633	0.042532	0.078263	0.048073	0.178465
AFR18	Afrino	0.631175	1.00E-04	0.122592	0.200116	0.028789	0.017227
MER51	Merino	0.630781	1.00E-04	0.130244	0.227855	1.00E-04	0.010921
MER56	Merino	0.629576	0.008866	0.151938	0.207519	1.00E-04	0.002002
MER47	Merino	0.627683	0.017275	0.135895	0.20109	0.006859	0.011198
AFR10	Afrino	0.626981	0.003917	0.149534	0.190496	0.013513	0.015558
MER62	Merino	0.626036	0.009698	0.127321	0.20478	1.00E-04	0.032065
MER32	Merino	0.620545	0.008892	0.136189	0.206748	1.00E-04	0.027526
AFR25	Afrino	0.618359	0.022911	0.133075	0.181433	0.017895	0.026327
MER52	Merino	0.61601	0.014832	0.138227	0.20667	1.00E-04	0.024161
AFR2	Afrino	0.608825	0.021939	0.114379	0.20481	0.029573	0.020474
SAM9	SAMerino	0.607779	0.024913	0.141675	0.201278	1.00E-04	0.024254
MER38	Merino	0.605824	0.014887	0.118046	0.225592	1.00E-04	0.035551
AFR6	Afrino	0.604495	0.017337	0.142208	0.186344	0.021708	0.027908
MER28	Merino	0.599241	0.024342	0.158292	0.179254	1.00E-04	0.03877
MER21	Merino	0.59778	0.004572	0.140779	0.214641	1.00E-04	0.042128
MER42	Merino	0.596298	1.00E-04	0.154312	0.227589	1.00E-04	0.0216
AFR33	Afrino	0.596009	0.021863	0.148052	0.184692	0.031649	0.017735
MER34	Merino	0.594317	1.00E-04	0.139303	0.227968	1.00E-04	0.038211
AFR19	Afrino	0.593154	0.006815	0.137789	0.194449	1.00E-04	0.067694
AFR14	Afrino	0.59306	0.014965	0.157739	0.196355	0.00857	0.029312
WCS88	WCS-affected	0.585159	0.076482	0.087993	0.093183	0.00093	0.156253
WCS110	WCS-affected	0.58466	0.087521	0.008466	0.001872	0.111091	0.206391
MER25	Merino	0.581524	0.044656	0.132641	0.188475	0.019278	0.033426
WCS1	WCS-affected	0.568988	0.0394	0.134081	0.195439	1.00E-04	0.061992
MER39	Merino	0.567273	0.007737	0.141288	0.190158	1.00E-04	0.093445
MER57	Merino	0.562653	0.046033	0.148913	0.193684	0.00783	0.040887
MER23	Merino	0.561739	0.003798	0.160113	0.218807	1.00E-04	0.055443
MER31	Merino	0.560295	0.018729	0.154001	0.20204	1.00E-04	0.064835
WCS6	WCS-affected	0.558297	0.016509	0.150178	0.215255	1.00E-04	0.059661
MER35	Merino	0.553923	0.028556	0.137117	0.213289	1.00E-04	0.067015
AFR34	Afrino	0.552419	0.059332	0.147814	0.184652	1.00E-04	0.055683
AFR16	Afrino	0.552033	0.045464	0.15274	0.177623	0.016417	0.055725
AFR15	Afrino	0.550521	0.042775	0.131658	0.228004	0.000582	0.04646
MER19	Merino	0.548526	0.026713	0.145051	0.191922	1.00E-04	0.087689
MER27	Merino	0.542715	0.029407	0.143852	0.221266	1.00E-04	0.062661
AFR22	Afrino	0.535821	0.056838	0.14542	0.173849	0.021075	0.066998
AFR3	Afrino	0.52917	0.071111	0.161947	0.170844	0.013869	0.053058
AFR26	Afrino	0.528326	0.064006	0.132195	0.203302	0.001989	0.070182
DOH19	DohneMerino	0.527593	0.022464	0.020051	0.076433	0.059995	0.293464
CTRL14	Unaffected	0.519714	0.02648	0.004764	1.00E-04	0.069261	0.379681
WCS64	WCS-affected	0.516042	0.022405	1.00E-04	1.00E-04	0.073384	0.387969
AFR7	Afrino	0.510002	0.075858	0.145626	0.166049	0.031625	0.07084
AFR11	Afrino	0.505749	0.0539	0.148551	0.177289	0.029158	0.085354

DOR10	Dorper	0.475657	0.064347	0.001459	0.029356	0.069764	0.359419
MER17	Merino	0.466901	0.092019	0.149927	0.165684	0.017544	0.107924
DOH44	DohneMerino	0.447579	0.02908	0.107375	0.138473	0.054216	0.223276
DOR11	Dorper	0.425226	0.027928	0.051996	0.165491	0.039563	0.289795
DOH16	DohneMerino	0.349882	0.069164	0.15836	0.214478	0.05895	0.149167
WCS53	WCS-affected	0.327505	0.1157	0.227228	0.205082	0.01893	0.105555
MER16	Merino	1.00E-04	0.9995	1.00E-04	1.00E-04	1.00E-04	1.00E-04
MER36	Merino	1.00E-04	0.9995	1.00E-04	1.00E-04	1.00E-04	1.00E-04
MER44	Merino	1.00E-04	0.9995	1.00E-04	1.00E-04	1.00E-04	1.00E-04
MER46	Merino	1.00E-04	0.9995	1.00E-04	1.00E-04	1.00E-04	1.00E-04
MER50	Merino	1.00E-04	0.9995	1.00E-04	1.00E-04	1.00E-04	1.00E-04
MER60	Merino	1.00E-04	0.9995	1.00E-04	1.00E-04	1.00E-04	1.00E-04
SAMM8	SAMuttonMerino	1.00E-04	0.9995	1.00E-04	1.00E-04	1.00E-04	1.00E-04
WCS3	WCS-affected	1.00E-04	0.997954	1.00E-04	1.00E-04	0.001646	1.00E-04
MER55	Merino	1.00E-04	0.996465	1.00E-04	1.00E-04	0.003135	1.00E-04
MER29	Merino	1.00E-04	0.995989	1.00E-04	0.003611	1.00E-04	1.00E-04
MER58	Merino	1.00E-04	0.99259	1.00E-04	0.007011	1.00E-04	1.00E-04
MER48	Merino	1.00E-04	0.99223	1.00E-04	1.00E-04	0.007371	1.00E-04
MER63	Merino	1.00E-04	0.990473	1.00E-04	0.002928	1.00E-04	0.006299
AFR5	Afrino	0.023052	0.967147	0.002031	1.00E-04	1.00E-04	0.00757
MER12	Merino	0.008721	0.966991	1.00E-04	1.00E-04	0.023988	1.00E-04
AFR9	Afrino	0.010906	0.964132	1.00E-04	0.006737	0.007447	0.010679
MER53	Merino	0.02904	0.952676	1.00E-04	0.012724	0.00536	1.00E-04
AFR1	Afrino	0.03236	0.949948	0.002999	0.014493	1.00E-04	1.00E-04
AFR24	Afrino	0.017355	0.949055	0.013232	0.020158	1.00E-04	1.00E-04
MEATM13	MeatMaster	0.015756	0.94769	0.004807	0.012284	0.01936	0.000102
AFR40	Afrino	0.024124	0.945076	1.00E-04	0.020512	1.00E-04	0.010089
MER40	Merino	1.00E-04	0.944664	1.00E-04	1.00E-04	0.054936	1.00E-04
AFR36	Afrino	0.019595	0.939563	0.020921	0.019722	1.00E-04	1.00E-04
AFR28	Afrino	0.043164	0.933488	0.003105	0.020043	1.00E-04	1.00E-04
MEATM19	MeatMaster	0.031596	0.924491	0.00834	0.035373	1.00E-04	1.00E-04
AFR17	Afrino	0.019452	0.923777	0.015963	0.01519	0.002673	0.022945
AFR20	Afrino	0.022949	0.90229	0.021608	0.047133	1.00E-04	0.00592
MEATM6	MeatMaster	0.029948	0.8881	0.036687	0.008564	0.027434	0.009268
MEATM18	MeatMaster	0.034517	0.887025	0.004591	0.011367	0.041946	0.020553
WCS4	WCS-affected	0.03164	0.887013	0.018131	0.011551	0.047521	0.004146
SAMM9	SAMuttonMerino	0.050251	0.881271	0.009216	0.035042	0.022956	0.001265
AFR32	Afrino	0.05896	0.87555	0.021094	0.034466	1.00E-04	0.009831
MEATM25	MeatMaster	0.015062	0.87504	0.01189	0.045827	0.016531	0.03565
MER14	Merino	0.037994	0.870444	0.016215	0.038449	0.028189	0.00871
MER41	Merino	0.04644	0.869862	0.008651	0.050018	0.018918	0.006112
MER22	Merino	0.069771	0.869352	0.019853	0.040825	1.00E-04	1.00E-04
AFR45	Afrino	0.003955	0.868977	0.042613	1.00E-04	0.070064	0.014291
MEATM22	MeatMaster	0.042395	0.865388	0.0278	0.037349	0.005782	0.021286

DOR22	Dorper	0.016552	0.865124	0.01942	0.036782	0.038096	0.024026
DOR17	Dorper	0.061368	0.86197	0.027477	0.046849	0.001455	0.000882
MER26	Merino	0.045451	0.861068	0.028035	0.012264	0.042043	0.01114
MER37	Merino	0.043549	0.858683	0.028592	0.015595	0.023398	0.030183
MEATM7	MeatMaster	0.044748	0.857624	0.017887	0.040308	0.004753	0.034681
AFR13	Afrino	0.077736	0.855213	0.001434	0.059008	0.003887	0.002722
BHP2	BlackheadPersian	0.027493	0.853153	0.023381	0.039536	0.029033	0.027405
MER15	Merino	0.063085	0.850252	0.016027	0.063029	1.00E-04	0.007508
DOR16	Dorper	0.066701	0.846481	0.033671	0.043316	0.009269	0.000561
MER6	Merino	0.056266	0.845747	0.007402	0.050314	0.034965	0.005306
MER13	Merino	0.077467	0.835856	0.014134	0.038634	0.018816	0.015094
MER33	Merino	0.059082	0.830515	0.041942	0.028583	0.039778	1.00E-04
MER24	Merino	0.036723	0.829332	0.034355	0.023726	0.054205	0.02166
MEATM1	MeatMaster	0.069913	0.823444	0.026107	0.055692	0.009895	0.014949
AFR49	Afrino	0.048238	0.81192	0.027927	0.036609	0.061555	0.013751
MER20	Merino	0.069129	0.797429	0.02291	0.086628	1.00E-04	0.023805
MEATM12	MeatMaster	0.067278	0.776622	0.05657	0.046698	0.02739	0.025441
BHP6	BlackheadPersian	0.0604	0.766075	0.044806	0.050605	0.050332	0.027783
MER18	Merino	0.095287	0.749867	0.054451	0.072263	1.00E-04	0.028031
AFR41	Afrino	0.081087	0.73855	0.056607	0.076647	0.019605	0.027504
BHP13	BlackheadPersian	0.062768	0.736959	0.053877	0.079711	0.005617	0.061067
MEATM46	MeatMaster	0.070348	0.73672	0.068034	0.070618	0.021749	0.032531
MEATM31	MeatMaster	0.093084	0.710276	0.061159	0.084592	1.00E-04	0.050789
MEATM44	MeatMaster	0.091394	0.683454	0.040263	0.089826	0.047438	0.047625
MER30	Merino	0.108819	0.582218	0.058343	0.08634	0.116589	0.04769
MEATM30	MeatMaster	0.104253	0.493403	0.100917	0.090531	0.142096	0.0688
MEATM35	MeatMaster	0.087595	0.492437	0.111312	0.08778	0.142037	0.078839
MEATM42	MeatMaster	0.133926	0.481263	0.091587	0.090472	0.12067	0.082083
AFR50	Afrino	0.125433	0.479281	0.085632	0.107141	0.124238	0.078275
MER9	Merino	0.127291	0.468395	0.101653	0.109169	0.128318	0.065173
MEATM40	MeatMaster	0.12484	0.464686	0.10777	0.092489	0.137809	0.072407
MER3	Merino	0.142861	0.46109	0.090581	0.108551	0.115982	0.080936
BHP3	BlackheadPersian	0.142272	0.454857	0.09438	0.125725	0.109256	0.07351
BHP10	BlackheadPersian	0.143547	0.45374	0.087917	0.129767	0.102995	0.082033
BHP7	BlackheadPersian	0.136238	0.445499	0.101768	0.132258	0.110612	0.073626
BHP14	BlackheadPersian	0.143533	0.43989	0.113684	0.129914	0.074034	0.098945
DOH20	DohneMerino	1.00E-04	1.00E-04	0.9995	1.00E-04	1.00E-04	1.00E-04
WCS76	WCS-affected	1.00E-04	1.00E-04	0.9995	1.00E-04	1.00E-04	1.00E-04
WCS113	WCS-affected	1.00E-04	1.00E-04	0.9995	1.00E-04	1.00E-04	1.00E-04
WCS118	WCS-affected	1.00E-04	1.00E-04	0.9995	1.00E-04	1.00E-04	1.00E-04
WCS116	WCS-affected	1.00E-04	1.00E-04	0.997215	1.00E-04	0.002385	1.00E-04
DOH11	DohneMerino	1.00E-04	1.00E-04	0.997111	0.002039	0.00055	1.00E-04
WCS112	WCS-affected	1.00E-04	0.020565	0.979035	1.00E-04	1.00E-04	1.00E-04
WCS100	WCS-affected	1.00E-04	0.015419	0.973649	0.00676	0.003972	1.00E-04

NAM3	NamakwaAfrikaner	0.011908	1.00E-04	0.971816	0.004747	0.011329	1.00E-04
DOH25	DohneMerino	0.016282	0.006963	0.96456	1.00E-04	0.011995	1.00E-04
WCS80	WCS-affected	1.00E-04	1.00E-04	0.963817	0.035784	1.00E-04	1.00E-04
WCS75	WCS-affected	1.00E-04	0.009131	0.960465	0.00871	0.021494	1.00E-04
DOH4	DohneMerino	0.000193	1.00E-04	0.952788	0.037077	0.009742	1.00E-04
CTRL59	Unaffected	1.00E-04	0.022539	0.949884	1.00E-04	0.017341	0.010036
CTRL60	Unaffected	1.00E-04	0.041488	0.945214	1.00E-04	1.00E-04	0.012998
CTRL30	Unaffected	1.00E-04	0.033164	0.944792	1.00E-04	0.012156	0.009689
NAM5	NamakwaAfrikaner	0.030972	1.00E-04	0.940133	0.017746	0.010949	1.00E-04
WCS74	WCS-affected	1.00E-04	0.017621	0.933792	0.03924	0.009147	1.00E-04
WCS114	WCS-affected	0.002028	0.021433	0.933702	0.042637	1.00E-04	1.00E-04
CTRL55	Unaffected	1.00E-04	0.025552	0.932819	1.00E-04	0.024273	0.017156
CTRL57	Unaffected	1.00E-04	0.045675	0.929367	1.00E-04	0.010734	0.014024
WCS91	WCS-affected	1.00E-04	0.022484	0.928968	0.030354	0.011013	0.007081
DOH14	DohneMerino	1.00E-04	0.011324	0.926668	0.061708	1.00E-04	1.00E-04
DOH22	DohneMerino	1.00E-04	0.011065	0.925509	0.035943	0.023876	0.003507
WCS85	WCS-affected	1.00E-04	0.010602	0.925508	0.049064	0.013666	0.00106
DOH48	DohneMerino	1.00E-04	0.048359	0.924304	1.00E-04	0.006409	0.020728
WCS115	WCS-affected	0.017664	0.002952	0.922947	0.034537	0.021801	1.00E-04
CTRL62	Unaffected	0.026142	0.01877	0.922913	1.00E-04	0.005078	0.026996
WCS83	WCS-affected	0.008157	0.020588	0.921983	0.033164	1.00E-04	0.016008
WCS84	WCS-affected	1.00E-04	0.049462	0.917762	1.00E-04	0.032476	1.00E-04
NAM6	NamakwaAfrikaner	0.022368	1.00E-04	0.916258	0.052347	0.008827	1.00E-04
CTRL58	Unaffected	0.003313	0.018618	0.915015	0.013905	0.022367	0.026783
CTRL31	Unaffected	0.024326	0.042946	0.910166	0.0091	0.013361	1.00E-04
CTRL53	Unaffected	1.00E-04	0.01103	0.909549	0.010768	0.028894	0.039659
CTRL24	Unaffected	0.000268	0.024306	0.908993	0.006768	0.046492	0.013173
DOH47	DohneMerino	0.009685	0.033823	0.908984	1.00E-04	0.024467	0.022941
WCS93	WCS-affected	0.011412	1.00E-04	0.907024	0.066197	0.015167	1.00E-04
NAM1	NamakwaAfrikaner	1.00E-04	0.002687	0.902498	0.067893	0.024146	0.002677
WCS87	WCS-affected	1.00E-04	0.005544	0.898878	0.063276	0.032103	1.00E-04
WCS89	WCS-affected	1.00E-04	0.000149	0.895921	0.077701	0.026028	1.00E-04
NAM2	NamakwaAfrikaner	1.00E-04	1.00E-04	0.895233	0.082876	0.021591	1.00E-04
WCS79	WCS-affected	1.00E-04	0.003388	0.890157	0.106155	1.00E-04	1.00E-04
NAM4	NamakwaAfrikaner	1.00E-04	0.028219	0.890027	1.00E-04	0.081454	1.00E-04
WCS81	WCS-affected	0.002005	0.030662	0.889889	0.061665	0.015679	1.00E-04
DOH49	DohneMerino	0.011662	0.033172	0.888322	0.002773	0.028351	0.035721
DOR13	Dorper	0.006666	0.037002	0.883254	0.041883	0.021387	0.009809
WCS82	WCS-affected	0.005179	0.008747	0.87602	0.077316	0.032639	1.00E-04
CTRL21	Unaffected	1.00E-04	0.045235	0.875148	0.022963	0.023746	0.032807
DOH5	DohneMerino	0.012862	0.00871	0.874266	0.093435	0.010627	1.00E-04
WCS98	WCS-affected	0.005428	0.033361	0.867345	0.070303	0.023462	1.00E-04
WCS119	WCS-affected	1.00E-04	0.020967	0.860757	0.067974	0.050101	1.00E-04
WCS73	WCS-affected	1.00E-04	0.009071	0.856095	0.128348	1.00E-04	0.006286

CTRL4	Unaffected	1.00E-04	0.032671	0.853415	0.064461	0.040097	0.009257
DOH2	DohneMerino	0.031299	1.00E-04	0.851078	0.078467	0.034339	0.004717
CTRL34	Unaffected	0.031312	0.028177	0.849314	0.066256	0.010647	0.014295
CTRL3	Unaffected	1.00E-04	0.011282	0.847993	0.11629	0.024235	1.00E-04
DOH24	DohneMerino	0.00551	0.008762	0.84698	0.115445	0.023203	1.00E-04
CTRL5	Unaffected	0.03779	0.011687	0.82538	0.091107	0.033937	1.00E-04
WCS123	WCS-affected	0.001715	0.021583	0.821471	0.115734	0.032002	0.007495
CTRL2	Unaffected	1.00E-04	0.028136	0.820754	0.115333	0.024063	0.011614
DOH46	DohneMerino	0.003301	0.021831	0.819096	0.093185	0.040878	0.021709
WCS92	WCS-affected	0.02051	0.02723	0.812058	0.112615	0.010501	0.017086
WCS120	WCS-affected	1.00E-04	0.0163	0.804025	0.118219	0.061256	1.00E-04
CTRL16	Unaffected	0.045539	0.020159	0.80229	0.087419	0.026587	0.018006
WCS121	WCS-affected	1.00E-04	1.00E-04	0.793397	0.152188	0.054115	1.00E-04
CTRL56	Unaffected	0.005855	0.046485	0.786258	0.088967	0.043527	0.028908
CTRL22	Unaffected	0.022893	0.030981	0.778942	0.122069	0.037713	0.007403
CTRL63	Unaffected	0.013057	0.040811	0.778231	0.086815	0.064694	0.016392
MER91	Merino	0.00094	0.081883	0.76378	0.005682	0.137725	0.00999
MER10	Merino	0.079916	1.00E-04	0.745892	0.039126	0.01435	0.120615
WCS124	WCS-affected	1.00E-04	0.009277	0.74072	0.227065	0.022738	1.00E-04
MER11	Merino	0.104194	1.00E-04	0.73353	0.062548	1.00E-04	0.099528
MEATM36	MeatMaster	0.107271	1.00E-04	0.729439	0.049443	0.013822	0.099925
MEATM41	MeatMaster	0.085217	1.00E-04	0.716252	0.064352	0.031981	0.102098
MER7	Merino	0.098878	1.00E-04	0.704168	0.063866	0.021113	0.111874
WCS103	WCS-affected	1.00E-04	0.035649	0.702886	0.178099	0.066789	0.016477
DOH12	DohneMerino	0.05027	1.00E-04	0.69922	0.250211	1.00E-04	1.00E-04
CTRL19	Unaffected	0.004595	0.05718	0.699206	0.129912	0.069147	0.039959
MEATM11	MeatMaster	0.093283	1.00E-04	0.69588	0.096948	1.00E-04	0.113689
MER4	Merino	0.086036	1.00E-04	0.694985	0.084815	0.041046	0.093018
DOR6	Dorper	0.009777	0.033253	0.693107	0.184328	0.06326	0.016276
MEATM43	MeatMaster	0.053595	0.01732	0.689793	0.014794	0.103961	0.120538
MEATM17	MeatMaster	0.078328	1.00E-04	0.68942	0.090916	0.034236	0.107
DOH23	DohneMerino	1.00E-04	0.006915	0.68155	0.226718	0.084616	1.00E-04
DOH37	DohneMerino	0.121283	1.00E-04	0.681295	0.180974	1.00E-04	0.016248
WCS122	WCS-affected	1.00E-04	0.027087	0.679474	0.212136	0.057649	0.023554
DOH34	DohneMerino	0.060424	1.00E-04	0.678958	0.202166	0.013027	0.045326
MEATM45	MeatMaster	0.113235	1.00E-04	0.677068	0.093219	1.00E-04	0.116277
MER5	Merino	0.12983	1.00E-04	0.676665	0.087264	1.00E-04	0.106041
CTRL50	Unaffected	0.055474	0.018843	0.671889	0.230501	1.00E-04	0.023193
MEATM24	MeatMaster	0.099845	0.000435	0.663514	0.11899	0.008042	0.109174
WCS86	WCS-affected	0.007181	0.044488	0.638254	0.228345	0.048533	0.033199
DOR21	Dorper	0.090754	1.00E-04	0.626916	0.119398	0.050409	0.112423
CTRL44	Unaffected	0.012194	0.010107	0.62112	0.27595	0.066775	0.013855
CTRL18	Unaffected	0.043886	0.014462	0.618209	0.235415	0.054569	0.033459
MER1	Merino	0.06305	0.056647	0.617348	0.005624	0.146207	0.111124

CTRL33	Unaffected	0.109987	1.00E-04	0.615978	0.2726	1.00E-04	0.001235
CTRL1	Unaffected	0.000794	0.03033	0.612656	0.262224	0.065192	0.028805
MEATM5	MeatMaster	0.113659	1.00E-04	0.611214	0.158147	0.016024	0.100856
DOH50	DohneMerino	0.06293	0.012661	0.601061	0.276696	0.007951	0.038701
DOH21	DohneMerino	1.00E-04	0.037487	0.598699	0.256696	0.106919	1.00E-04
WCS117	WCS-affected	0.025042	0.02133	0.591348	0.335697	0.021644	0.004939
DOH45	DohneMerino	0.035845	0.023166	0.584773	0.244062	0.070323	0.041831
CTRL15	Unaffected	0.016064	0.041326	0.570047	0.26628	0.073801	0.032483
DOR15	Dorper	0.116001	0.006934	0.565521	0.129089	0.075847	0.106608
CTRL61	Unaffected	0.042503	0.019598	0.565341	0.309161	0.034916	0.028481
DOR5	Dorper	0.042999	0.026541	0.560107	0.281506	0.048418	0.040428
CTRL29	Unaffected	1.00E-04	0.024605	0.524302	0.282027	0.129151	0.039815
CTRL49	Unaffected	0.055642	0.034232	0.510694	0.278064	0.078321	0.043047
DOR3	Dorper	0.017354	0.032726	0.50311	0.383241	0.030123	0.033448
DOH10	DohneMerino	0.055409	0.01705	0.499279	0.391696	0.001715	0.034852
WCS95	WCS-affected	0.045798	0.023614	0.493813	0.396982	0.013507	0.026285
WCS78	WCS-affected	0.02193	0.028325	0.475666	0.460014	1.00E-04	0.013965
CTRL45	Unaffected	0.003087	0.044574	0.465831	0.380988	0.083455	0.022066
DOH38	DohneMerino	0.029416	0.059131	0.449851	0.326781	0.072267	0.062554
DOR2	Dorper	0.025447	0.066078	0.429098	0.360773	0.089477	0.029127
CTRL41	Unaffected	0.027758	0.029236	0.426595	0.421487	0.058281	0.036643
WCS94	WCS-affected	0.031914	0.013895	0.425838	0.5156	1.00E-04	0.012654
DOH1	DohneMerino	0.070227	0.030222	0.418658	0.403165	0.043166	0.034562
CTRL17	Unaffected	0.012373	0.074968	0.415929	0.316564	0.122494	0.057673
CTRL47	Unaffected	1.00E-04	0.097697	0.41556	0.281206	0.174535	0.030903
AFR27	Afrino	1.00E-04	0.050874	0.379479	0.135002	0.357546	0.076998
BHP5	BlackheadPersian	1.00E-04	0.061877	0.375296	0.146868	0.360948	0.054912
AFR31	Afrino	1.00E-04	0.065724	0.372489	0.151472	0.331502	0.078713
AFR39	Afrino	1.00E-04	0.053773	0.360981	0.16403	0.35753	0.063586
AFR35	Afrino	1.00E-04	0.049012	0.358032	0.187222	0.331247	0.074388
BHP12	BlackheadPersian	1.00E-04	0.032627	0.358018	0.182593	0.346525	0.080137
AFR43	Afrino	1.00E-04	0.067824	0.356601	0.148608	0.348271	0.078595
AFR48	Afrino	1.00E-04	0.076731	0.351738	0.156385	0.346958	0.068088
WCS46	WCS-affected	0.218467	0.208393	0.254789	0.084045	0.114952	0.119355
NAM10	NamakwaAfrikaner	1.00E-04	1.00E-04	1.00E-04	0.9995	1.00E-04	1.00E-04
WCS52	WCS-affected	1.00E-04	1.00E-04	1.00E-04	0.9995	1.00E-04	1.00E-04
WCS54	WCS-affected	1.00E-04	1.00E-04	1.00E-04	0.9995	1.00E-04	1.00E-04
WCS60	WCS-affected	1.00E-04	1.00E-04	1.00E-04	0.9995	1.00E-04	1.00E-04
WCS63	WCS-affected	1.00E-04	1.00E-04	1.00E-04	0.9995	1.00E-04	1.00E-04
NAM9	NamakwaAfrikaner	1.00E-04	1.00E-04	0.013915	0.985685	1.00E-04	1.00E-04
WCS61	WCS-affected	1.00E-04	1.00E-04	1.00E-04	0.970265	0.029335	1.00E-04
MER64	Merino	1.00E-04	1.00E-04	1.00E-04	0.968384	0.031216	1.00E-04
WCS59	WCS-affected	1.00E-04	1.00E-04	0.005512	0.950486	0.043702	1.00E-04
WCS57	WCS-affected	0.007708	0.002325	0.025675	0.950302	0.013891	1.00E-04

WCS62	WCS-affected	1.00E-04	1.00E-04	0.050248	0.949352	1.00E-04	1.00E-04
WCS58	WCS-affected	1.00E-04	1.00E-04	0.046353	0.948449	0.004898	1.00E-04
SAM4	SAMerino	1.00E-04	0.005054	0.04258	0.938542	0.013625	1.00E-04
WCS48	WCS-affected	0.007609	1.00E-04	0.032451	0.908151	0.051589	1.00E-04
WCS55	WCS-affected	1.00E-04	0.016072	1.00E-04	0.904677	0.078951	1.00E-04
SAM7	SAMerino	1.00E-04	0.015962	0.027904	0.897132	0.052996	0.005907
WCS56	WCS-affected	1.00E-04	1.00E-04	0.075077	0.894304	0.030319	1.00E-04
NAM8	NamakwaAfrikaner	1.00E-04	1.00E-04	0.058657	0.888009	0.053034	1.00E-04
WCS51	WCS-affected	1.00E-04	1.00E-04	0.052212	0.879701	0.055552	0.012336
WCS49	WCS-affected	1.00E-04	0.029106	0.067344	0.874131	0.029219	1.00E-04
WCS50	WCS-affected	0.000962	0.035089	0.025773	0.860621	0.077456	1.00E-04
WCS47	WCS-affected	1.00E-04	0.003314	0.093231	0.834571	0.064692	0.004092
SAM3	SAMerino	1.00E-04	0.034369	0.059557	0.82317	0.078842	0.003962
SAM2	SAMerino	0.017184	0.010922	0.094444	0.820479	0.051469	0.005502
SAM5	SAMerino	1.00E-04	0.005981	0.079591	0.818551	0.095677	1.00E-04
WCS44	WCS-affected	0.023116	0.021917	0.066062	0.818456	0.070349	1.00E-04
SAM6	SAMerino	1.00E-04	0.025116	0.044302	0.817154	0.113228	1.00E-04
WCS45	WCS-affected	0.005562	0.004541	0.123001	0.792629	0.05968	0.014588
DOH3	DohneMerino	0.009463	0.016506	0.062808	0.772112	0.101738	0.037373
SAM1	SAMerino	0.005394	0.018324	0.187049	0.740699	0.033419	0.015116
SAM8	SAMerino	0.051239	0.011444	0.125149	0.734535	0.067248	0.010385
DOR4	Dorper	1.00E-04	0.029981	0.081767	0.732456	0.108152	0.047544
CTRL27	Unaffected	0.039756	0.060266	0.117273	0.708321	0.055657	0.018727
CTRL32	Unaffected	0.023388	0.049716	0.126913	0.685368	0.088579	0.026037
WCS77	WCS-affected	1.00E-04	0.058187	0.131245	0.667636	0.108756	0.034077
CTRL26	Unaffected	0.030819	0.039924	0.147502	0.662407	0.085586	0.033762
NAM7	NamakwaAfrikaner	1.00E-04	0.039109	0.137504	0.65837	0.16094	0.003978
DOR12	Dorper	0.002375	0.030344	0.196055	0.623114	0.091748	0.056364
CTRL40	Unaffected	0.01106	0.068394	0.143598	0.59097	0.141846	0.044131
CTRL48	Unaffected	0.024857	0.04734	0.205069	0.587522	0.089123	0.04609
CTRL25	Unaffected	1.00E-04	0.069829	0.055936	0.585764	0.226945	0.061427
CTRL51	Unaffected	0.028868	0.053574	0.18078	0.578118	0.116741	0.041919
CTRL38	Unaffected	0.027181	0.044894	0.249911	0.573157	0.057924	0.046933
CTRL36	Unaffected	1.00E-04	0.054023	0.249357	0.571084	0.085443	0.039992
CTRL52	Unaffected	0.054359	0.045016	0.214517	0.568577	0.072963	0.044568
DOR1	Dorper	0.004977	0.085607	0.117458	0.567023	0.169299	0.055637
CTRL54	Unaffected	0.010271	0.06701	0.151319	0.552061	0.157988	0.061351
DOH7	DohneMerino	0.05656	0.011701	0.372862	0.549915	1.00E-04	0.008862
CTRL35	Unaffected	1.00E-04	0.030589	0.322215	0.545637	0.060168	0.041291
WCS90	WCS-affected	0.029842	0.042002	0.276109	0.540844	0.087147	0.024056
CTRL43	Unaffected	0.000678	0.065016	0.198552	0.529271	0.160023	0.046461
DOH6	DohneMerino	0.050104	0.009433	0.390942	0.528323	1.00E-04	0.021098
DOH40	DohneMerino	0.03311	0.024209	0.362239	0.520935	0.026001	0.033506
WCS72	WCS-affected	0.064612	0.028174	0.365111	0.51752	0.011282	0.013302

DOH9	DohneMerino	0.06818	0.009607	0.40023	0.508432	0.00579	0.007761
CTRL42	Unaffected	0.028658	0.057018	0.238585	0.505038	0.137798	0.032902
DOH39	DohneMerino	0.065684	0.070918	0.281011	0.49795	0.040983	0.043454
CTRL46	Unaffected	0.012832	0.040762	0.288072	0.497496	0.090581	0.070257
DOH8	DohneMerino	0.067498	0.027301	0.393743	0.49214	1.00E-04	0.019218
DOH36	DohneMerino	0.042061	0.03921	0.338094	0.490754	0.051906	0.037976
DOR7	Dorper	0.193508	1.00E-04	0.266233	0.462767	1.00E-04	0.077292
DOH35	DohneMerino	0.089258	0.033734	0.364594	0.459819	0.011745	0.04085
CTRL28	Unaffected	0.101085	0.013739	0.343908	0.453334	0.037498	0.050435
DOH41	DohneMerino	0.056442	0.069252	0.354724	0.449519	0.043253	0.026809
CTRL20	Unaffected	0.082406	0.046096	0.403677	0.442347	0.002705	0.022769
CTRL37	Unaffected	0.020322	0.045168	0.338403	0.433022	0.099215	0.06387
WCS105	WCS-affected	0.0022	0.032869	0.402119	0.424892	0.086389	0.051531
AFR21	Afrino	1.00E-04	1.00E-04	1.00E-04	1.00E-04	0.9995	1.00E-04
AFR30	Afrino	1.00E-04	1.00E-04	1.00E-04	1.00E-04	0.9995	1.00E-04
AFR42	Afrino	1.00E-04	1.00E-04	1.00E-04	1.00E-04	0.9995	1.00E-04
AFR51	Afrino	1.00E-04	1.00E-04	1.00E-04	1.00E-04	0.9995	1.00E-04
BHP4	BlackheadPersian	1.00E-04	1.00E-04	1.00E-04	1.00E-04	0.9995	1.00E-04
BHP8	BlackheadPersian	1.00E-04	1.00E-04	1.00E-04	1.00E-04	0.9995	1.00E-04
DOR14	Dorper	1.00E-04	1.00E-04	1.00E-04	1.00E-04	0.9995	1.00E-04
MEATM8	MeatMaster	1.00E-04	1.00E-04	1.00E-04	1.00E-04	0.9995	1.00E-04
MEATM10	MeatMaster	1.00E-04	1.00E-04	1.00E-04	1.00E-04	0.9995	1.00E-04
MEATM14	MeatMaster	1.00E-04	1.00E-04	1.00E-04	1.00E-04	0.9995	1.00E-04
MEATM20	MeatMaster	1.00E-04	1.00E-04	1.00E-04	1.00E-04	0.9995	1.00E-04
MEATM23	MeatMaster	1.00E-04	1.00E-04	1.00E-04	1.00E-04	0.9995	1.00E-04
MEATM29	MeatMaster	1.00E-04	1.00E-04	1.00E-04	1.00E-04	0.9995	1.00E-04
MEATM2	MeatMaster	1.00E-04	0.000775	1.00E-04	1.00E-04	0.998825	1.00E-04
BHP11	BlackheadPersian	1.00E-04	0.001164	1.00E-04	1.00E-04	0.998436	1.00E-04
DOR18	Dorper	1.00E-04	1.00E-04	1.00E-04	0.015354	0.984246	1.00E-04
MEATM34	MeatMaster	1.00E-04	0.011779	0.009985	1.00E-04	0.977936	1.00E-04
MEATM4	MeatMaster	1.00E-04	0.010789	1.00E-04	0.016282	0.964486	0.008243
MEATM39	MeatMaster	1.00E-04	0.016862	1.00E-04	0.025312	0.957526	1.00E-04
MEATM48	MeatMaster	0.011322	1.00E-04	1.00E-04	0.051614	0.930615	0.006249
DOR20	Dorper	1.00E-04	1.00E-04	0.008589	0.046723	0.921178	0.02331
MEATM26	MeatMaster	1.00E-04	0.002845	0.016476	0.072246	0.879221	0.029112
AFR46	Afrino	0.012542	0.015211	0.005005	0.078712	0.878999	0.009531
AFR38	Afrino	0.02227	1.00E-04	0.032738	0.092422	0.828482	0.023988
MEATM16	MeatMaster	1.00E-04	0.027337	0.027394	0.108803	0.796722	0.039644
MEATM27	MeatMaster	0.04354	0.176275	0.041554	0.169885	0.460017	0.108728
BHP1	BlackheadPersian	0.004389	0.181066	0.086072	0.158716	0.452792	0.116965
AFR4	Afrino	0.03708	0.175833	0.076726	0.164367	0.437897	0.108097
AFR23	Afrino	0.061769	0.131963	0.034745	0.22202	0.432601	0.116901
MEATM3	MeatMaster	0.028794	0.177314	0.076105	0.167373	0.428535	0.121879
MEATM21	MeatMaster	0.06812	0.128475	0.025735	0.24963	0.420617	0.107423

BHP9	BlackheadPersian	0.041696	0.155588	0.071933	0.187623	0.420226	0.122935
AFR12	Afrino	0.079618	0.13039	0.028081	0.243261	0.414188	0.104462
AFR44	Afrino	0.04413	0.134107	0.045152	0.249933	0.409333	0.117346
DOR19	Dorper	0.074128	0.121305	0.050975	0.249648	0.398172	0.105771
MEATM9	MeatMaster	0.077725	0.105999	0.044094	0.26252	0.3929	0.116762
AFR8	Afrino	0.062843	0.108959	0.054637	0.262695	0.38835	0.122517
MEATM15	MeatMaster	0.078506	0.125881	0.051503	0.252288	0.380616	0.111206
AFR47	Afrino	0.021463	0.057382	0.271459	0.218723	0.337406	0.093567
WCS43	WCS-affected	1.00E-04	1.00E-04	1.00E-04	1.00E-04	1.00E-04	0.9995
SAMM1	SAMuttonMerino	0.003023	1.00E-04	1.00E-04	1.00E-04	0.00317	0.993507
WCS7	WCS-affected	1.00E-04	0.006439	1.00E-04	1.00E-04	1.00E-04	0.993161
SAMM2	SAMuttonMerino	1.00E-04	1.00E-04	1.00E-04	1.00E-04	0.00858	0.99102
WCS30	WCS-affected	1.00E-04	1.00E-04	1.00E-04	1.00E-04	0.008957	0.990643
WCS21	WCS-affected	0.010522	1.00E-04	1.00E-04	1.00E-04	1.00E-04	0.989078
SAMM7	SAMuttonMerino	1.00E-04	1.00E-04	1.00E-04	1.00E-04	0.01316	0.98644
WCS36	WCS-affected	1.00E-04	1.00E-04	1.00E-04	0.014158	1.00E-04	0.985442
WCS28	WCS-affected	1.00E-04	0.007653	0.006748	0.002457	1.00E-04	0.982942
WCS38	WCS-affected	0.001032	0.011043	1.00E-04	1.00E-04	0.007915	0.97981
WCS34	WCS-affected	1.00E-04	1.00E-04	1.00E-04	1.00E-04	0.021023	0.978577
WCS33	WCS-affected	1.00E-04	0.008216	1.00E-04	0.007549	0.011465	0.97257
WCS41	WCS-affected	1.00E-04	0.000871	0.000388	0.002769	0.023546	0.972326
WCS32	WCS-affected	1.00E-04	0.001869	0.025998	1.00E-04	0.002664	0.969269
WCS24	WCS-affected	1.00E-04	0.011318	0.007652	1.00E-04	0.012381	0.96845
WCS20	WCS-affected	1.00E-04	1.00E-04	0.01067	0.009038	0.013629	0.966463
WCS18	WCS-affected	0.018367	1.00E-04	1.00E-04	0.014958	1.00E-04	0.966375
WCS35	WCS-affected	0.00565	0.002743	1.00E-04	0.02219	0.004324	0.964994
WCS13	WCS-affected	1.00E-04	0.00541	1.00E-04	0.016933	0.017455	0.960002
WCS12	WCS-affected	1.00E-04	1.00E-04	1.00E-04	0.042274	1.00E-04	0.957326
WCS31	WCS-affected	0.012918	0.004938	1.00E-04	0.022405	0.008522	0.951118
SAMM3	SAMuttonMerino	0.021685	1.00E-04	0.026723	1.00E-04	0.002786	0.948607
WCS23	WCS-affected	0.008516	0.012126	1.00E-04	0.026773	0.007529	0.944957
SAMM4	SAMuttonMerino	0.018945	1.00E-04	0.005374	1.00E-04	0.031826	0.943655
WCS25	WCS-affected	0.04626	1.00E-04	0.00501	1.00E-04	0.008632	0.939899
WCS19	WCS-affected	0.015721	0.015751	1.00E-04	0.015911	0.014102	0.938415
WCS14	WCS-affected	0.05726	1.00E-04	0.004089	1.00E-04	0.000807	0.937644
WCS37	WCS-affected	0.003746	0.010173	1.00E-04	0.03013	0.021743	0.934109
WCS42	WCS-affected	1.00E-04	0.007285	1.00E-04	0.033899	0.028904	0.929712
WCS39	WCS-affected	0.039318	0.003283	1.00E-04	0.014169	0.026288	0.916842
WCS29	WCS-affected	0.022856	1.00E-04	1.00E-04	0.064642	1.00E-04	0.912202
WCS9	WCS-affected	0.039162	0.005338	1.00E-04	0.019243	0.029346	0.90681
SAMM6	SAMuttonMerino	0.042118	0.013437	1.00E-04	0.014553	0.024622	0.905169
WCS16	WCS-affected	0.032811	0.006773	1.00E-04	0.043605	0.017459	0.899252
WCS22	WCS-affected	0.054432	0.004506	1.00E-04	0.026212	0.020688	0.894062
WCS17	WCS-affected	0.031629	0.010628	0.003202	0.017821	0.044554	0.892166

WCS40	WCS-affected	0.063321	0.013655	0.004042	0.013178	0.015366	0.890438
WCS10	WCS-affected	0.048395	0.009612	0.010137	0.019523	0.022682	0.889652
WCS11	WCS-affected	0.087474	1.00E-04	0.003453	0.0219	1.00E-04	0.886974
SAMM5	SAMuttonMerino	0.039315	0.002306	0.020038	0.03981	0.026562	0.87197
WCS27	WCS-affected	0.064896	0.007072	0.008624	0.026164	0.023923	0.869322
WCS26	WCS-affected	0.080401	0.005143	0.010244	0.013337	0.0227	0.868175
WCS15	WCS-affected	0.09662	0.005503	0.001329	0.02815	0.011227	0.857172
SAM10	SAMerino	0.045746	0.033882	0.018954	0.023304	0.025531	0.852584
WCS8	WCS-affected	0.112894	1.00E-04	0.026471	0.022427	1.00E-04	0.838009
MEATM47	MeatMaster	0.167332	0.060862	0.182907	0.07914	1.00E-04	0.509659
MEATM37	MeatMaster	0.167011	0.082118	0.17296	0.073471	0.017401	0.48704
AFR37	Afrino	0.191268	0.071394	0.150895	0.116797	1.00E-04	0.469545
MEATM33	MeatMaster	0.235073	0.052176	0.142383	0.11585	1.00E-04	0.454419
MER2	Merino	0.239162	0.059481	0.16999	0.090608	1.00E-04	0.44066
AFR29	Afrino	0.245244	0.055646	0.149341	0.110338	1.00E-04	0.43933
MEATM32	MeatMaster	0.243278	0.043349	0.177288	0.098833	1.00E-04	0.437152
MER8	Merino	0.282258	0.023398	0.162566	0.131828	1.00E-04	0.399851
MEATM38	MeatMaster	0.255722	0.069036	0.159871	0.123355	1.00E-04	0.391916
MEATM28	MeatMaster	0.260247	0.053602	0.167469	0.126782	1.00E-04	0.391801
